# Protective Role of Acetylsalicylic Acid in Experimental *Trypanosoma cruzi* Infection: Evidence of a 15-epi-Lipoxin A_4_-Mediated Effect

**DOI:** 10.1371/journal.pntd.0002173

**Published:** 2013-04-18

**Authors:** Alfredo Molina-Berríos, Carolina Campos-Estrada, Natalia Henriquez, Mario Faúndez, Gloria Torres, Christian Castillo, Sebastián Escanilla, Ulrike Kemmerling, Antonio Morello, Rodrigo A. López-Muñoz, Juan D. Maya

**Affiliations:** 1 Molecular and Clinical Pharmacology Program, ICBM, Faculty of Medicine, University of Chile, Santiago, Chile; 2 Centro de Investigación Biomédica, Facultad de Medicina, Universidad Diego Portales, Santiago, Chile; 3 Departamento de Farmacia, Facultad de Química. Pontificia Universidad Católica de Chile, Santiago, Chile; 4 Anatomy and Developmental Biology Program, ICBM, Faculty of Medicine, University of Chile, Santiago, Chile; Instituto de Investigaciones Biotecnológicas, Argentina

## Abstract

Chagas' disease, produced by *Trypanosoma cruzi*, affects more than 8 million people, producing approximately 10,000 deaths each year in Latin America. Migration of people from endemic regions to developed countries has expanded the risk of infection, transforming this disease into a globally emerging problem. PGE_2_ and other eicosanoids contribute to cardiac functional deficits after infection with *T. cruzi*. Thus, the inhibition of host cyclooxygenase (COX) enzyme emerges as a potential therapeutic target. *In vivo* studies about the effect of acetylsalicylic acid (ASA) upon *T. cruzi* infection are controversial, and always report the effect of ASA at a single dose. Therefore, we aimed to analyze the effect of ASA at different doses in an *in vivo* model of infection and correlate it with the production of arachidonic acid metabolites. ASA decreased mortality, parasitemia, and heart damage in *T. cruzi* (Dm28c) infected mice, at the low doses of 25 and 50 mg/Kg. However, this effect disappeared when the high ASA doses of 75 and 100 mg/Kg were used. We explored whether this observation was related to the metabolic shift toward the production of 5-lipoxygenase derivatives, and although we did not observe an increase in LTB_4_ production in infected RAW cells and mice infected, we did find an increase in 15-epi-LXA_4_ (an ASA-triggered lipoxin). We also found high levels of 15-epi-LXA_4_ in *T. cruzi* infected mice treated with the low doses of ASA, while the high ASA doses decreased 15-epi-LXA_4_ levels. Importantly, 15-epi-LXA_4_ prevented parasitemia, mortality, and cardiac changes *in vivo* and restored the protective role in the treatment with a high dose of ASA. This is the first report showing the production of ASA-triggered lipoxins in *T. cruzi* infected mice, which demonstrates the role of this lipid as an anti-inflammatory molecule in the acute phase of the disease.

## Author Summary

Chagas' disease is an infection produced by the parasite *Trypanosoma cruzi*. This pathology is endemic in Latin America and has become a public health issue in some non-endemic countries like the USA, Spain and Australia. There is no curative treatment against Chagas' disease. NSAIDs, like aspirin, have been assayed as drugs with therapeutic potential in Chagas' disease, but the studies about this issue show contradictory results; also, the mechanism involved in aspirin effect is not yet clear. In this study, we explore a broad range of doses the protective role of aspirin. We found that aspirin has a therapeutic effect at low doses, an effect that disappears when doses are increased. This phenomenon correlates with the presence of 15-epi-LXA_4_, a molecule known as an “aspirin-triggered lipoxin,” which increases at low doses of aspirin, and decreases when aspirin dose is increased. 15-epi-LXA_4_ has been related with the anti-inflammatory effect of aspirin; in this setting, we found that 15-epi-LXA_4_ is able to decrease the cardiac inflammation and others parameters related with Chagas' disease. Finally, we present the first study that shows that the protective effect of aspirin on Chagas' disease could be mediated by the synthesis of 15-epi-LXA_4_.

## Introduction

American Trypanosomiasis (Chagas' disease) is a parasitic illness caused by the flagellate protozoan *Trypanosoma cruzi* [1]. The area covered by this disease starts in the south of the United States and continues to the central area of Chile and Argentina. It has been present in America for 9,000 years [2]. In Latin America, Chagas' disease affects more than 8 million people, causing approximately 10,000 deaths each year, which is higher than malaria in the Americas, and covers 89% of the deaths caused by tropical-cluster diseases [3]. In addition, there is an annual productivity loss of US$1.2 billion due to Chagas' disease in the 7 endemic countries [4]. Furthermore, the migration of people from endemic regions to developed countries has expanded the risk of infection, especially through blood transfusions and organ transplants. As a consequence, there are currently immigrant infected populations in Japan, Australia, Spain, and in the United States, transforming this disease into an emerging global problem [5]. In addition, the impact of Chagas' disease in U.S. has been recently compared to the first years of the beginning of the VIH/AIDS epidemic [6].

The acute phase of Chagas' disease is characterized by immunosuppression induced by *T. cruzi* to evade the host immune response. This immunosuppressive state is mediated by prostaglandins [7], [8] and cytokines, such as transforming growth factor-β (TGF-β) [9]. Increased circulating levels of prostaglandin E_2_ (PGE_2_) [10], thromboxane A_2_ (TXA_2_), and prostaglandin F_2α_ (PGF_2α_) have been reported in mice infected with *T. cruzi* [11], and during the acute phase, macrophages and spleen cells from *T. cruzi*-infected mice produce high levels of PGE_2_ [10]. Thus, as PGE_2_ and other eicosanoids might contribute to cardiac remodeling and other cardiac functional deficits after infection with *T. cruzi*, the inhibition of the host cyclooxygenase (COX) enzyme emerges as a potential therapeutic target.

In infected BALB/c mice, treatment with aspirin, indomethacin or celecoxib decreases parasitemia and delays mortality [7], [12]. However, some gaps remains in the literature data, since all assays described have been carried out with fixed doses of the COX inhibitor studied.

Recently, the effect of ASA has been associated, at least in part, to a metabolic switch towards a pathway linked to the acetylation of the COX-2 isoenzyme. This acetylation enables COX-2 to synthetize other lipid products derived from AA, some of them with anti-inflammatory properties [13]. These metabolic products have been called “ASA-triggered lipoxins” (ATLs). Correspondingly, ASA-triggered 15-epi-Lipoxin-A_4_ (15-epi-LXA_4_) has been described as an anti-inflammatory lipid able to inhibit IL-6, TNF-α and IL-8 production, as well as NFκB, ERK1/2 and p38 activation [14].

In this report, we explore the relation between the protective role of ASA and the synthesis of 15-epi-LXA_4_. In first place, we show that ASA treatment has a protective effect in *T.cruzi*-infected mice. However, this effect disappears with the higher doses employed. In addition, we found that infected mice treated with the effective ASA doses (25 or 50 mg/Kg/day) produce 15-epi-LXA_4_, whereas higher doses inhibit its production. Based upon these data, we propose that the protective role of ASA in experimentally *T. cruzi*-infected mice is related to the production of 15-epi-LXA_4_.

## Materials and Methods

### Ethic statement

All animal handling protocols were performed according to the “Guide for the Care and Use of Laboratory Animals”, from the National Institute of Health, USA [15], and approved by the Institutional Ethical Committee at the Faculty of Medicine, University of Chile (Protocol CBA# 0277 FMUCH), associated to FONDECYT-Chile grant number 1090078.

### In vivo infection model

Adult male BALB/c mice (20–25 g) were obtained from the Animal Facility at the Faculty of Medicine, University of Chile. Animals were first infected intraperitoneally with 30,000 *T. cruzi* blood trypomastigotes (Dm28c strain). Afterwards, animals were randomized to receive the different treatments. *T. cruzi* infection was followed daily by parasitemia through direct microscopic visualization of circulating trypomastigotes from peripheral blood, as previously described [16].

### Treatment administration

Acetylsalicylic acid (Sigma, USA) was given diluted in the drinking water at concentrations that ranged from 19.5 to 390 mg/L, to achieve final doses from 5 to 100 mg/Kg/day, based in the observation that mice drank 6.4 mL of water daily. The water was available *ad libitum*. The bottles were replaced every morning with fresh water or drug solution, and the residual volume of water in bottles was measured to assure that the mice drank the intended water volume [17], [18]. The treatment was initiated 48 hours after parasite inoculation, for 20 days at the doses indicated in each figure. As a measure of the pharmacological effect of orally administered aspirin, we determined the bleeding time in mice. To do this, we made a cut in the tail tip and bleeding was evaluated with filter paper every 15 seconds. These determinations were made at the day 10 post-infection (p.i.). (Table 1). 5(S),6(R),15(R)-Lipoxin A_4_ (Cayman Chemicals, USA), was diluted daily in fresh PBS and administered i.p. for 10 days, starting at the fourth day p.i., using the doses indicated in each figure. Controls received PBS exclusively. Mortality rate was recorded daily.

**Table 1:** Effect of oral-administered ASA over bleeding time in *T. cruzi* infected mice.

	Bleeding time (s)^a^	
	Mean	SD
Control	129.3	41.6
ASA 5 mg/Kg/day	257.9^b^	68.1
ASA 50 mg/Kg/day	258.6^b^	38.9

a *T. cruzi*-infected mice were treated with ASA 5 or 50 mg/Kg/day, diluted in drink water. Bleeding time was measured at day 10 p.i.

b p<0.001 compared with control, measured by ANOVA.

### Cardiac histopathological analysis

Hearts were extracted at the moment of death from those mice dead before the end point. Surviving mice were euthanized at day 20 p.i. and their hearts were extracted. Hearts were longitudinally sectioned to further analysis by histopathology and qPCR. Samples were fixed in 10% formaldehyde 0.1 M phosphate buffer (pH 7.3) for 24 h, dehydrated in alcohol, clarified in xylene, and embedded in paraffin. 5 µm sections were obtained and stained with hematoxylin-eosin for routine histopathological analysis as well as to evaluate the presence of *T. cruzi* amastigote nests and inflammation of the myocardium [19]. The automated morphometric analysis was performed by methodology developed by us, using the public domain software ImageJ (ver 1.46). The photographs of at least four nonconsecutive slides of tissue from different mice were assessed. The contrast of each photograph was increased automatically. To facilitate visualization of cell nuclei, the color channels (red, blue and green) were split. To isolate nuclei marks, the red channel was transformed in a binary image, using the “threshold” tool. To separate particles corresponding to adjacent nuclei, the “watershed” filter was applied. To exclude amastigote nuclei, only particles over 50 pixels were included in the particle count. Results of this procedure were compared with those obtained from manual count to verify the accuracy of the automatic counting. There was less than 10% variation between manual and automated count. To eliminate bias in the application of the methodology, the procedure was kept as a “macro” tool to apply the count automatically without intervention of the researcher.

### Real-time PCR

Hearts from infected animals treated with ASA or 15-epi-LXA_4_ were homogenized, and DNA was isolated using the Wizard Genomic DNA Purification Kit (Promega, USA), following manufacturer's instructions. DNA was quantified through 280 nm absorbance measurements using a Varioskan spectrophotometer (Thermo Scientific, USA). Parasite DNA quantification was performed using the primers TCZ-F (5′-GCTCTTGCCCACAMGGGTGC-3′) and TCZ-R (5′-CCAAGCAGCGGATAGTTCAGG-3′), designed to amplify a 195 bp Satellite-DNA sequence of *T. cruzi* [20]. We used the TNFα-5241 (5′-TCCCTCTCATCAGTTCTATGGCCCA-3′) and TNFα-5411 (5′-CAGCAAGCATCTATGCACTTAGACCCC-3′) primers, which amplify a 170 bp sequence of the *Mus musculus* TNF-α gene as loading control [20], [21]. PCR amplifications were carried out in the 7300 Real Time PCR system (Applied Biosystems, USA). All reactions were performed using 10 ng of DNA and using the SensiMix SYBR Hi-Rox Kit (Bioline, UK) at a final volume of 20 µL. For both primer pairs, the thermal cycles consisted of one 10 min step of polymerase at 95°C, followed by 40 cycles of 15 s at 95°C, 15 s at 60°C, and 30 s at 72°C. Fluorescence was measured at the end of each amplification cycle. Finally, the melting curve was performed between 60 and 95°C.

### Cell culture and in vitro infection model

RAW 264.7 cells (murine macrophages, ATCC number CRL-2922) were cultured at a density of 250,000 cells/cm^2^, in RPMI 1640 medium, supplemented with 5% fetal bovine serum, in humidified air with 5% CO_2_, at 37°C. RAW cells were infected with *T. cruzi* trypomastigotes (Dm28c strain) at a 3∶1 ratio (trypomastigote∶RAW cell). Trypomastigotes were allowed to infect cells for 24 hours, after which supernatant was extracted. Cells were then washed twice with sterile PBS (pH 7.4) to extract extracellular trypomastigotes.

### Western blot determination of COX isoforms and 5-LOX

For protein isolation, RAW cells were washed with PBS, scraped, and lysed by sonication. Hearts from control or infected mice were homogenized at 4°C in a Potter-Elvejem homogenizer. All samples were homogenized in lysis buffer at pH 8, containing Tris 0.01 µM, SDS 1%, and protease inhibitor cocktail (Complete Mini EDTA-free, Roche, USA). Total protein was quantified using the bicinchoninic acid method, using the BCA Pierce kit (Pierce Biotechnology, USA), following manufacturer instructions. For electrophoresis, extracted proteins were mixed with loading buffer (10% SDS, 50% glycerol, 0.5 M Tris, 0.1% bromphenol blue, and 1 M dithiothreitol, pH 6.8), and 40 µg (from the *in vitro* samples) or 30 µg (from the *in vivo* samples) of total protein was loaded into 8% polyacrylamide gels. After, proteins were transferred to nitrocellulose membranes, and were blocked overnight with BSA (3% in PBST 0.05%). After three washes with PBST, membranes were incubated overnight at 4°C with primary polyclonal antibodies against COX-1 (ab59964, Abcam, UK), COX-2 (ab52237, Abcam, UK) or 5-LOX (ab59341, Abcam, UK), diluted in PBST. Membranes were washed with PBST, and incubated with a HRP-conjugated anti-rabbit-IgG secondary antibody (ab6721, Abcam, UK) for 1 hour. Afterwards, membranes were washed with PBST, and developed through chemiluminescence using the Pierce ECL Western Blotting Substrate (Pierce Biotechnology, USA). After developing, membranes were incubated on stripping solution (62.5 mM Tris-HCl (pH 6.8), 2% SDS and 50 mM of mercaptoethanol) at 50°C for 30 minutes. Membranes were blocked and incubated with a primary antibody against β-actin (ab3280, Abcam, UK), overnight at 4°C. We used a polyclonal anti-mouse-IgG Ab (ab6728, Abcam, UK) HRP-conjugated as secondary antibody. Developed films were scanned, and band densitometry analysis was performed using ImageJ software.

### PGE_2_, LTB_4_ and 15-epi-LXA_4_ determination

For PGE_2_ and LTB_4_ *in vitro* determinations, 10^6^ RAW cells were cultured in 24 well plates, and exposed to 3×10^6^ trypomastigotes (Dm28c strain). After 24 hours, supernatant was removed and infected cells were washed twice with PBS (pH 7.4), and treated with ASA at different concentrations. After 48 hours of treatment, PGE_2_ and LTB_4_ were assayed in the supernatant, using the Prostaglandin E_2_ EIA Kit and Leukotriene B_4_ EIA Kit (Cayman Chemicals, USA) immunoassays, following manufacturer instructions. LTB_4_ from animal plasma was measured using the Parameter(r) Kit (R&D Systems, USA), following manufacturer's instructions. 15-epi-LXA_4_ was assayed in the same model *in vitro* and *in vivo*, using the LXA_4_-15epi BioAssay ELISA Kit (USBiological, USA). For *in vivo* determinations, plasma from infected mice was collected after 10 days p.i., and assayed directly following manufacturer instructions.

### Statistical analysis

For all experiments, the statistical significance was established at p<0.05. Results represent mean ± SD of triplicates. All statistical analyses were performed using GraphPad Prism (5.0) software. Normal distribution of data was assessed using D'Agostino-Pearsons analysis. One- and two-way ANOVA analyses (with Tukey post-test) or non-parametric Kruskal-Wallis analyses (with Dunns post-test) were performed when required. For survival analysis, the log rank test was performed.

## Results

### Outcome of infected mice treated with ASA does not correlate with administered dose

**Figure 1:**
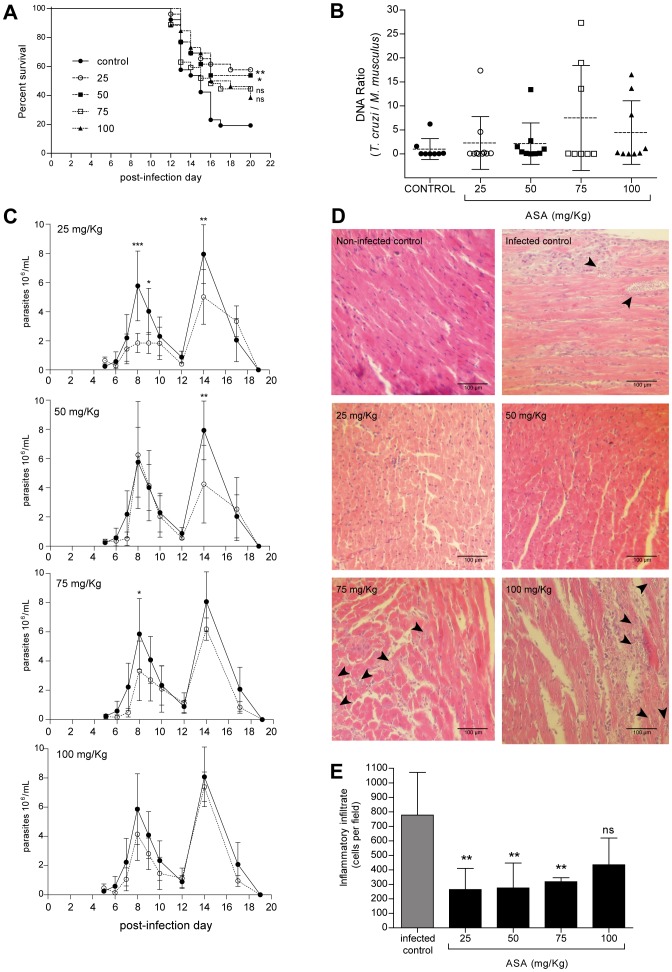
Effect of ASA on the outcome of experimental Chagas' disease. **A.** Survival of mice infected with *T. cruzi* (Dm28c strain), and treated with acetylsalicylic acid (ASA). The graph summarizes results obtained from four independent experiments with n = 6 each. *: p<0.01 and **: p<0.001 compared to control, by Kaplan-Meyer survival analysis; **ns**: no statistical differences. **B.** Real-time PCR analysis of hearts from infected mice treated with ASA. Hearts were extracted at the dead day, or on day 20 p.i. for survival mice. Each point represents one mouse. The discontinuous line indicates the mean of at least 8 mice. The results are expressed as the normalized ratio, relative to control. **C.** Parasitemia of *T. cruzi*-infected mice treated with ASA. The graphs show the effect of each dose compared to the control (to facilitate visual assessment, each dose is shown in a different panel). For each graph, black circles represent control groups, and white circles treated groups. Each measurement were obtained with n = 6 mice per group. *: p<0.05; **: p<0.01 and ***: p<0.001, compared to the same day control using two-way ANOVA. **D.** Histological examination of hearts from *T. cruzi* infected mice treated with acetylsalicylic acid (ASA). Representative histopathology of infected control mice and treated with ASA (25, 50, 75 or 100 mg/Kg/day). Hearts obtained at end point day were fixed in 10% formaldehyde and embedded in paraffin. Slides were stained with hematoxylin-eosin. Images are representative of at least three mice in each group. Black arrowheads indicate amastigote nests. **E.** Infiltrate quantification from histopathological analysis. Cells nuclei of infiltrate were quantified by using th ImageJ software. The graph shows the mean ± SD of at least five mice with three non-consecutive slides each. **: p<0.01 compared to control, calculated by two-way ANOVA and Tukey post-test.

We evaluated the effect of ASA on BALB/c mice infected with trypomastigotes of *T. cruzi* (Dm28c strain), at doses ranging from 5 to 100 mg/Kg. Figure 1A shows that treatment with 25 and 50 mg/Kg ASA significantly increased the survival of infected mice (p<0.01 and p<0.05, respectively). However, when the highest doses were used, (75 and 100 mg/Kg), the mortality rate was similar to that observed in the infected control. Similarly, lower ASA doses (<25 mg/Kg) did not affect mortality (data not shown). Thus, ASA impact upon survival was only observed at intermediate doses. Parasite DNA content was quantified by qPCR (Figure 1B), no significant differences were observed between the control and the ASA-treated groups.

ASA treatment was able to decrease parasitemia peaks at 25 and 50 mg/Kg (Figure 1C). In mice treated with ASA 25 mg/Kg the parasitemia was decreased significantly on days 8 and 14 p.i. (p<0.001 and p<0.01 respectively), while in the 50 mg/Kg group, parasitemia decreased significantly only on day 14 (p<0.01) (Figure 1C). Similarly, at 75 mg/Kg, the parasitemia peak on day 8 was decreased (p<0.05), although at a lower magnitude than that observed at 25 mg/Kg. No differences were observed at 100 mg/Kg when comparing to control. Finally, 5 and 10 mg/Kg of ASA did not show any differences when compared to control group (data not shown).

When we evaluated the cardiac structure of mice (Fig. 1D), we found that infected mice exhibited severe inflammatory infiltration, associated edema, and amastigote nests. At 25 mg/Kg ASA, there was less inflammation and edema, and heart tissue histology seemed normal. These protective effects disappeared when the ASA dose was increased, as seen in 100 mg/Kg ASA-treated mice, which showed more edema and inflammation than controls. In addition, amastigote nests were more evident in 75 and 100 mg/Kg treated mice. A quantification of inflammatory infiltrate (Figure 1E) showed that ASA 25, 50 and 75 mg/Kg decreased significantly the number of infiltrate cells. Although ASA 100 mg/Kg seems to have less infiltrate than control, this difference did not have statistical significance with the infected mice without treatment.

### Infected cells treated with ASA are able to synthetize 15-epi-LXA4

**Figure 2:**
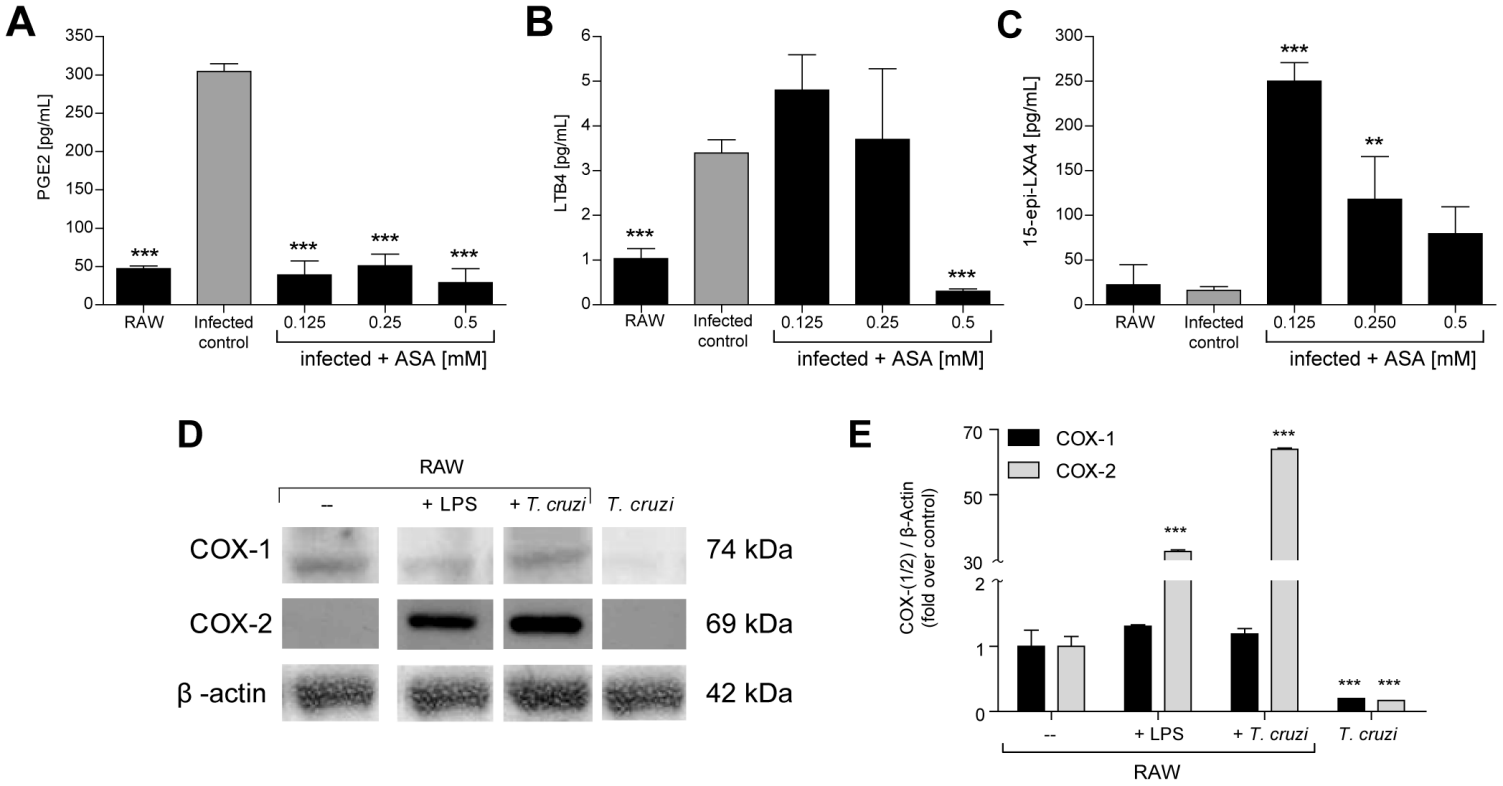
Effect of ASA upon eicosanoid production in RAW cells infected with *T. cruzi*. RAW 264.7 cells were incubated with *T. cruzi* trypomastigotes (Dm28c strain) for 24 hours. After, cells were treated with ASA at the indicated doses. **A.** PGE_2_ levels in the supernatants of infected and ASA treated RAW cell cultures at the indicated doses. **B.** LTB_4_ levels in the supernatants of infected and ASA treated RAW cell cultures at doses 0.125, 0.25 and 0.5 mM. **C.** 15-epi-LXA_4_ levels in supernatants of infected and ASA treated RAW cell cultures at doses 0.125, 0.25 and 0.5 mM. **D.** Western blot of COX-1 and COX-2 isoforms in RAW cells exposed to LPS 1 µg/mL (+LPS) or 3×10^6^ *T. cruzi* trypomastigotes (+*T cruzi*). Right lane corresponds to a crude extract of *T. cruzi* trypomastigote proteins. β-actin (botton panel) was used as loading control. **E.** Quantification of COX-1 and COX-2 blotting. All graphs are presented as the mean ± SD of triplicates, and are representative of at least two experiments. **: p<0.01 and ***: p<0.001 compared with its respective infected (panels A to C) or uninfected (panel E) control by two-way ANOVA.

We have previously reported that the effect of ASA upon *in vitro T. cruzi* infection is not reversed by exogenous PGE_2_ administration. Thus, we hypothesized that COX inhibitors, ASA in particular, have alternative mechanisms involved in this phenomenon. One possible mechanism could be ASA induction of a shift in the arachidonic acid metabolic pathway towards the production of 5-lipoxygenase derivatives [22]. Thus, we explored the variation in the metabolic pathway of AA, induced by ASA in an *in vitro* infection model. We assayed three concentrations of ASA, using 50% of our previously reported effect as a reference [23]. *T. cruzi* infection increased the PGE_2_ and LTB_4_ production in RAW 264.7 cells (Figure 2A and 2B). Correlated with the increase of PGE_2_ production, the COX-2 levels in RAW cells infected with *T. cruzi* also increased significantly (Figure 2D and 2E). As expected, ASA inhibited PGE_2_ synthesis at all tested concentrations (Figure 2A). In contrast, LTB_4_ production in ASA treated cells did not increase. Unexpectedly, we observed a significant low level of LTB_4_ in the 0.5 mM ASA treated cells, when compared with infected cells (Figure 2B). COX-2 acetylation by ASA modifies its activity, promoting the synthesis of 15-epi-LXA_4_, a lipid involved in the resolution of inflammation [24], [25]. Accordingly, we assessed the production of 15-epi-LXA_4_ in *T. cruzi* infected RAW cells treated with ASA. 15-epi-LXA_4_ production was significantly increased by ASA in these cells (Figure 2C). Interestingly, the production of 15-epi-LXA_4_ was inversely correlated to the concentration of ASA, reaching a level similar to the control at 0.5 mM ASA.

### Infected Balb/c mice treated with ASA have circulating 15-epi-LXA_4_

**Figure 3:**
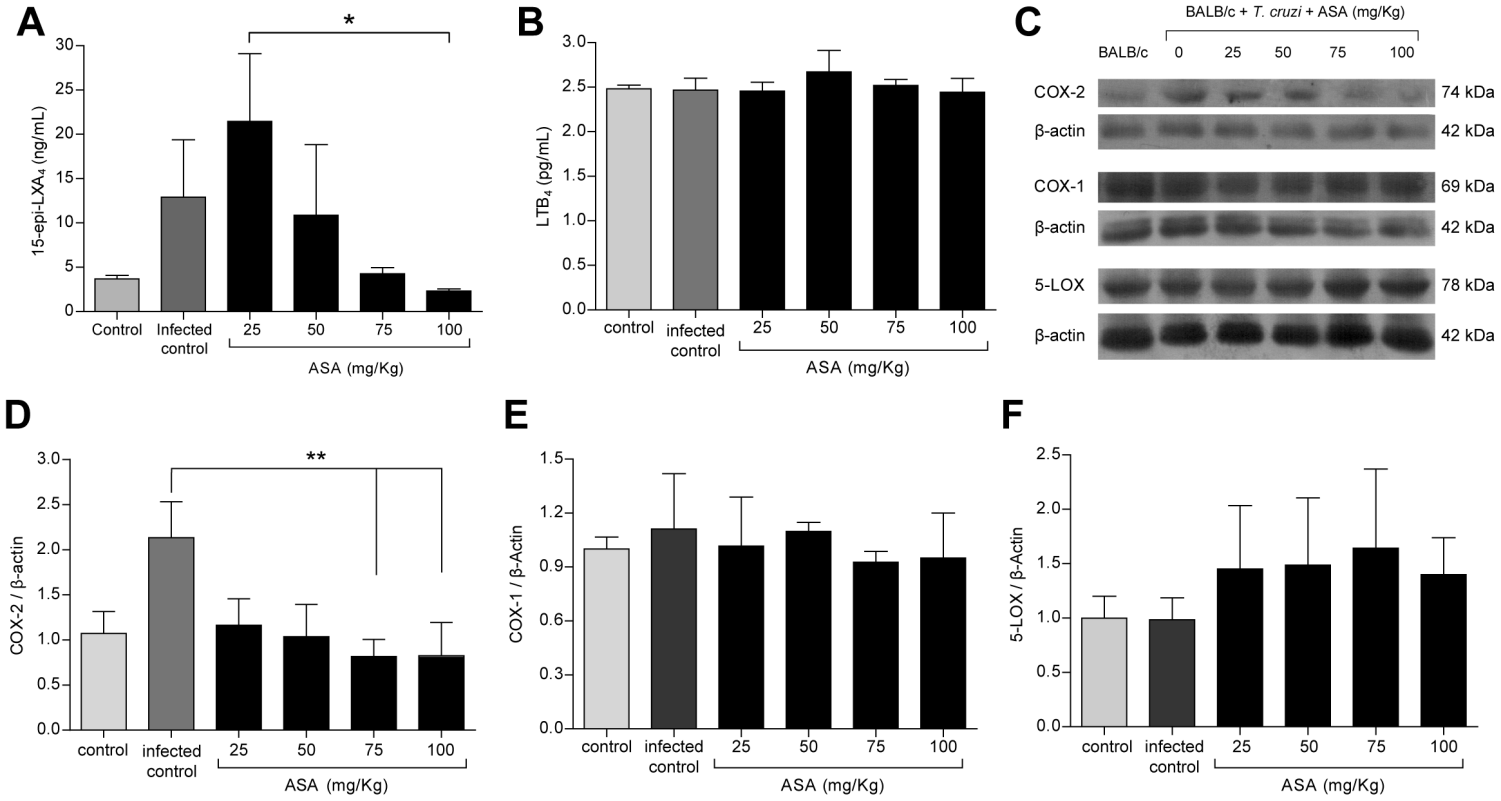
15-epi-LXA_4_ and LTB_4_ production on *T. cruzi* infected ASA treated mice. **A.** 15-epi-LXA_4_ plasma levels from infected mice treated with ASA on day 10 p.i. Graphs represent the mean ± SD of four mice per group, and are representative of two experiments. **B.** LTB_4_ plasma levels from infected mice treated with ASA on day 10 p.i. **C.** Western blots of COX-1, COX-2 and 5-LOX from cardiac tissue BALB/c mice on day 10 p.i. Gel photographs are representative of two experiments with at least n = 4 by group **D, E and F.** Quantification of relative intensity of the western blot bands from panel C. All Graphs represent the mean ± SD of at least four mice per group, and are representative of two experiments. *: p<0.05 and **: p<0.01 compared to uninfected control using Kruskal-Wallis test, followed by Dunns post-test.

Based on the previous *in vitro* results, we evaluated the generation of 15-epi-LXA_4_ and LTB_4_ in *T. cruzi*-infected mice. In agreement with *in vitro* data, low doses of ASA increased the circulating levels of 15-epi-LXA_4_, which decreased in a dose-depended manner (Figure 3A). Unpredictably, infection alone produced an increase in 15-epi-LXA_4_ in mice. This discrepancy in the 15-epi-LXA_4_ production observed between infected cells and mice may be due to the lack of cooperative systems in single mammalian cell model. In fact, coincubation of macrophages with polymorphonuclear neutrophils increases the production of 15-epi-LXA_4_ [26]. Therefore, it is expected that 15-epi-LXA_4_ production in mice to be more efficient than in RAW cells monoculture. ASA did not modify LTB_4_ production in infected mice (Figure 3B). Furthermore, *T. cruzi* infection did not change the basal levels of LTB_4_ (Figure 3B), contrary to what we observed in infected RAW cells (Figure 2B).

In the heart tissue of *T. cruzi* infected mice, COX-2 levels presented a two-fold increase compared with control (Figure 3C and 3D). These results are in agreement with previously reported data from immunohistochemical determinations [7]. Interestingly, the COX-2 levels appear to decrease with increasing ASA doses (Figure 3C and 3D), indeed the COX-2 levels in the 75 and 100 mg/Kg treated groups were statistically different with infected control. This data could explain why 15-epi-LXA_4_ levels are decreased in infected mice treated with ASA 75 or 100 mg/Kg. In addition, COX-1 and 5-LOX levels in cardiac tissue did not change with infection or treatment (Figures 3C, 3E and 3F). Thus, the changes in 15-epi-LXA_4_ production could be related with ASA effects on the COX-2 enzyme.

### 15-epi-LXA_4_ decreases parasite burden and cardiac inflammation in mice infected with *T. cruzi*

**Figure 4:**
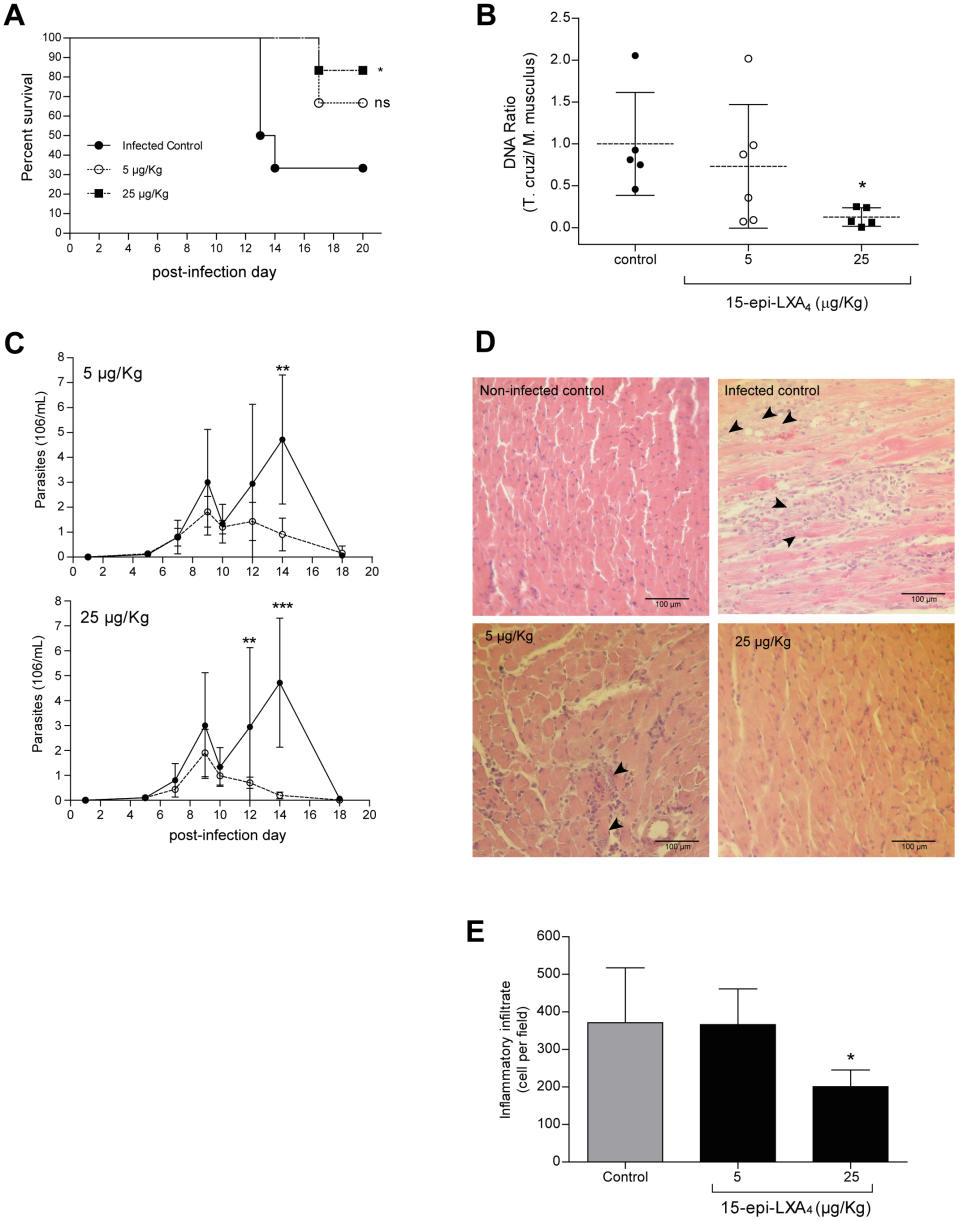
Effect of 15-epi-LXA4 on the outcome of experimental Chagas' disease. **A.** Survival of mice infected with *T. cruzi* (Dm28c strain) and treated with 15-epi-LXA_4_. Survival was recorded daily from day 1 p.i. Graph representative from two independent experiments (n = 6 each). *: p<0.01 as compared to the control, by Kaplan-Meyer survival analysis, ns: no statistical differences. **B.** Real-time PCR analysis of heart tissue from infected mice treated with 15-epi-LXA_4_. Hearts were extracted at the dead day, or on day 20 p.i. for survival mice. Each point represents one mouse. Discontinuous line indicates the mean from at least 5 mice. The results are expressed as the normalized ratio, relative to control *: p<0.05 compared to the control using Kruskal-Wallis test, followed by Dunns post-test. **C.** Parasitemia of *T. cruzi* infected mice treated with 15-epi-LXA_4_. To facilitate visual assessment, separate doses are showed in different panels. For each graph, black circles represent control groups, and white circles represent treated groups. All measurements were made with n = 6 mice per group. **: p<0.01 and ***: p<0.001, as compared to the same day control using two-way ANOVA. **D.** Histological examination of hearts from *T. cruzi* infected mice treated with 15-epi-LXA_4_. Representative histopathology of the infected control and of mice treated with 15-epi-LXA_4_ (5 or 25 mg/Kg/day). Hearts were extracted at the dead day, or on day 20 p.i. for survival mice. Hearts were fixed in 10% formaldehyde and embedded in paraffin. Slides were stained with hematoxylin-eosin. Black arrowheads indicate amastigote nests Images represent at least three mice in each group. **E.** Infiltrate quantification from histopathological analysis. Cells nuclei of infiltrate were quantified by using th ImageJ software. The graph shows the mean ± SD of at least five mice with three non-consecutive slides each. *: p<0.05 compared to control, calculated by two-way ANOVA and Tukey post-test.

Since ASA treatment can modify synthesis of 15-epi-LXA_4_ both *in vitro* and *in vivo*, we evaluated the effect of exogenous administration of 15-epi-LXA_4_ in the *T. cruzi* infection outcome in BALB/c mice. Infected mice were treated with 5 or 25 µg/Kg 15-epi-LXA_4_ according to previously reported schemes for other murine models [27]. 15-epi-LXA_4_ at 5 µg/Kg had no effect on the survival rate and cardiac parasite burden when quantified by relative DNA load (Figures 4A and 4B). In contrast, 15-epi-LXA_4_ at 25 µg/Kg significantly increased survival and decreased cardiac parasite load (Figure 4A and 4B). On the other hand, treatment with 5 and 25 µg/Kg 15-epi-LXA_4_ significantly decreased the parasitemia peaks observed on days 12 and 14 p.i. (Figure 4C). Cardiac histopathological analysis showed that 25 µg/Kg 15-epi-LXA_4_ decreased number of amastigote nests and the inflammatory infiltration (Figures 4D and 4E). Nevertheless, at 5 µg/Kg, focal inflammatory infiltration and amastigote nests persisted, as compared with untreated infected controls.

### 15-epi-lipoxin A_4_ restores the protective effect on *T. cruzi* infected-mice treated with high doses of ASA

**Figure 5:**
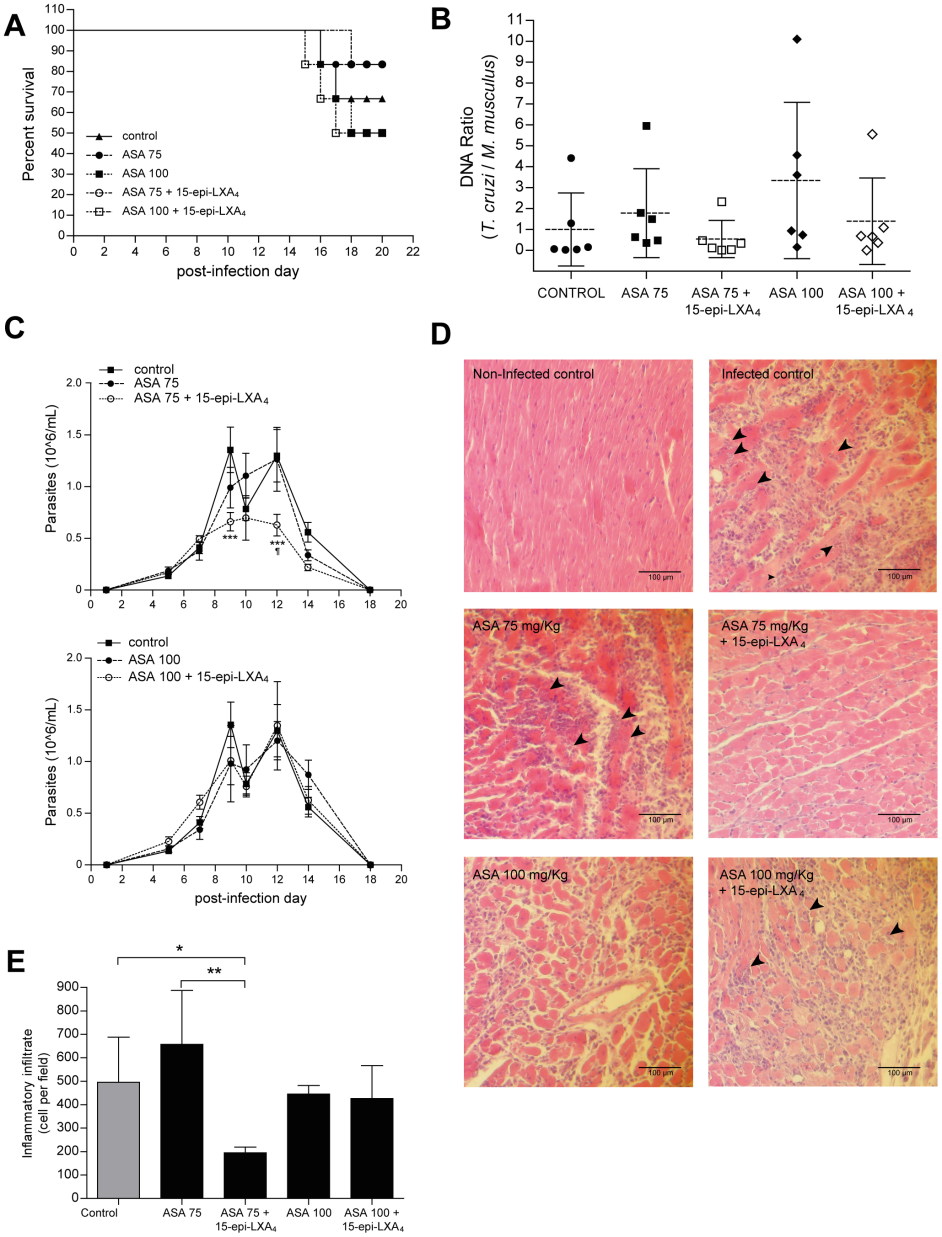
Effect of 15-epi-LXA_4_ on the outcome of experimental Chagas' disease in ASA-treated mice. **A.** Survival of mice infected with *T. cruzi* (Dm28c strain) treated with ASA (75 or 100 mg/Kg) with or withouh 15-epi-LXA_4_ (25 mg/Kg). Survival was recorded daily from day 1 p.i. Graph representative from two independent experiments (n = 6 each). **B.** Real-time PCR analysis of heart tissue from infected and treated mice. Hearts were extracted at the dead day, or on day 20 p.i. for survival mice. Each point represents one mouse. Discontinuous line indicates the mean from at least 5 mice. The results are expressed as the normalized DNA ratio, relative to control. **C.** Parasitemia of *T. cruzi-*infected and treated mice. To facilitate visual assessment, separate doses of ASA are showed in different panels. For each graph, black square represent control groups, black circles represents mice treated only with ASA and white circles represent the group treated with ASA and 15-epi-LXA_4_. All measurements were made with n = 6 mice per group. **: p<0.01 and ***: p<0.001, as compared to control and ¶: p<0.01 compared with corresponding ASA-treated group, calculated by two-way ANOVA. **D.** Histological examination of hearts from *T. cruzi* infected and treated mice. Hearts were extracted at the dead day, or on day 20 p.i. for survival mice. Hearts were fixed in 10% formaldehyde and embedded in paraffin. Slides were stained with hematoxylin-eosin. Black arrowheads indicate amastigote nests Images represent at least three mice in each group. **E.** Infiltrate quantification from histopathological analysis. Cells nuclei of infiltrate were quantified by using the ImageJ software. The graph shows the mean ± SD of at least five mice with three non-consecutive slides each. *: p<0.05 compared to control, and **:p<0.01 compared with ASA 75-treated group, calculated by two-way ANOVA and Tukey post-test.

Considering the above results, do high ASA doses lose its general protective effect on *T. cruzi* infected mice due to absence of 15-epi-LXA_4_ production? To answer this question, we administered 25 µg/Kg of 15-epi-LXA_4_ to *T. cruzi*-infected mice, treated with either 75 or 100 mg/Kg ASA. Although there were no significant effects of 15-epi-LXA_4_ on survival or cardiac parasite burden (Figures 5A and 5B), parasitemia, inflammatory infiltrate, and amastigote nests decreased when 15-epi-LXA_4_ was administered to the 75 mg/Kg ASA treated mice (Figures 5C, 5D and 5E). 15-epi-LXA_4_ did not produce effect in the 100 mg/Kg ASA treated-mice (Figure 5).

## Discussion

In this report, we showed that ASA decreased mortality, parasitemia, and heart damage in *T. cruzi* infected mice, at doses of 25 and 50 mg/Kg, doses below 25 mg/Kg did not alter the natural course of the disease in the infected mice, while mice treated with 75 and 100 mg/Kg/day of ASA, showed more intense symptoms. These results could be related to inhibition of COX-2 and consequent decrease in prostaglandin E_2_ production, with a metabolic shift to 5-LOX derivatives. However, as the expected increase in LTB_4_ production was not observed, the beneficial effect of this COX-2 inhibitor could be explained by the alternative production of 15-epi-LXA_4_. Indeed, this molecule prevented parasitemia, mortality and cardiac changes during the acute infection *in vivo*.

Arachidonic acid cascade research is an open field in the *T. cruzi*-host interaction studies. *In vivo* studies about the effect of COX inhibitors upon *T. cruzi* infection are controversial, because the reported results vary depending upon the mouse or *T. cruzi* strain used, the parasite inoculum, the type of inhibitor, or the therapeutic scheme [7], [8], [12], [28]–[31]. During early infection, treatment with aspirin or indomethacin dramatically increases parasitemia, and reduces the survival rate of *T. cruzi*-infected C57BL/6, C3H/HeN or CD-1 mice, all of which have been described as resistant to the acute infection [8], [29], [31], [32]. By the contrary, there is evidence the points out that COX inhibitors might improve parasitemia, heart damage and survival in *T. cruzi*-infected BALB/c mice [7], [12], [28]–[30]. This contradictory data might be explained by the variation in the production of arachidonic acid derivatives under different experimental conditions. Here, we found that low doses of ASA significantly improved the outcome of *T. cruzi*-infected mice and demonstrated that this effect is related with the production of 15-epi-LXA_4_, a metabolite of AA not previously associated with ASA effect on the Chagas' disease.

During acute infection, PGE_2_, TXA_2_, PGI_2_, and PGF_2α_ are increased [11], [30], [33]. PGE_2_ and TXA_2_ appear to modulate the host response against the parasite, and facilitate the shift to the chronic phase. Parasite-derived TXA_2_ is essential for parasite survival, regulation of amastigote replication during the acute stage, and modulation of the cardiac disease [33], [34]. Moreover, knocking out the host TXA_2_ receptor increases parasite load in cardiac tissue [34]. According to this, COX inhibition by ASA would be expected to produce a similar effect, but we found that aspirin treatment had a protective effect without decreasing parasite load in the heart tissue. Thus, at 25 mg/Kg ASA, cardiac tissue appeared normal; thus becoming evident that prostaglandins are not the only arachidonic acid metabolites involved in *T. cruzi* infection. In consequence, the parasitological protection provided by ASA, evidenced mainly by increase of survival and histological “normalization” in heart is probably, the result of a better immunological control provided by the metabolic shift toward the 15-epi-LXA4 production and the decrease in prostaglandins and thromboxane levels.

The role of 5-lipoxygenase and its metabolites in Chagas' disease has also been previously studied [35]–[37]. It has been reported that leukotrienes participate in parasitemia control, and are important for survival during the early acute phase of the infection, by facilitating the trypanocidal activity of phagocytic cells achieved through NO• production regulation [38]. This is in agreement with the observation that 5-lipoxygenase null-mutant mice infected with *T. cruzi* show a higher mortality rate than wild-type infected mice [36]. However, leukotriene participation in the overall Chagas' disease physiopathology process has not been described as pivotal, because there could be other mechanisms independent of leukotrienes through which an increase in NO• production could be achieved, as demonstrated by Panis and colleagues [36]. When we investigated the effect of ASA on RAW 264.7 cells, we found that prostaglandin E_2_ production was decreased, which indicates that COX-2 was inhibited. The shift of arachidonic acid metabolism toward leukotriene synthesis was ruled out, because ASA did not increase the LTB_4_ levels either *in vitro* or *in vivo*. Furthermore, *in vitro*, the level of LTB_4_ decreased as ASA concentration was increased, indicating that this drug also affected LTB_4_ metabolism at high doses.

In a *Toxoplasma gondii* infection model, treatment with ASA or LXA_4_ induces migration of dendritic cells (DCs) , and *in vivo* production of interleukin (IL)-12, through the induction of suppressor of cytokine signaling (SOCS)-2 expression, demonstrating a role of ATLs in parasitic infection control [39]. In agreement with those results, we showed that in *T. cruzi* infected mice, 15-epi-LXA_4_ decreased inflammatory infiltration in cardiac tissue and improve disease outcome. In addition, increased levels of ATLs in infected mice, and the decrease of parasitic load in cardiac tissue after treatment with 15-epi-LXA_4_, might be related to a potential role for this molecule in parasite contention, aside from its role in the resolution of the inflammation.

In the context of *T. cruzi* infection, there is production of 15-epi-LXA_4_, especially at low doses of ASA. This result agrees with the anti-inflammatory effect seen in humans when low doses of ASA were administered. This effect was related to ATL production [24]. In addition, a human trial showed that 15-epi-LXA4 production decreased when high doses of ASA were supplied [40]. Thus, the divergent effects observed with low and high doses of ASA observed in our model of murine Chagas' disease are supported by these results. However, the clinical utility of our findings might be limited as the effects are only found in one strain. Conversely, the findings of disease aggravation with ASA are not strain specific and may reflect the more likely clinical scenario in a genetically diverse population, such as the patients likely to encounter the disease

In conclusion, this is the first report showing the production of aspirin triggered lipoxins in *T. cruzi* infected mice suggesting a role of this lipid as an anti-inflammatory molecule in the acute phase of the disease.
