# Expression of Concern: Protective Role of Acetylsalicylic Acid in Experimental *Trypanosoma cruzi* Infection: Evidence of a 15-epi-Lipoxin A_4_-Mediated Effect

**DOI:** 10.1371/journal.pntd.0012471

**Published:** 2024-09-05

**Authors:** The PLOS Neglected Tropical Diseases Editors

Following the publication of this article [1], concerns were raised regarding results presented in Fig 2D. Specifically, the cropped β-actin bands for RAW -, RAW + LPS and *T*. *cruzi* (lanes 1, 2, and 4) appear similar despite representing different experimental conditions.

The corresponding author acknowledged that an error occurred during the preparation of this figure and stated that the original underlying images are no longer available. They stated that the underlying data for all other results remain available and can be provided on request.

They repeated the western blot experiment in Fig 2D and quantification in Fig 2E, and the results of this repeat experiment, underlying data, and revised protocol are provided with this notice in S1 and S2 Files.

There are discrepancies between the published results in Fig 2E and the repeat experiment, as COX-2 expression is lower in the Raw + LPS group than Raw + *T*. *cruzi* in the published graph, but higher than Raw + *T*. *cruzi* in the repeat experiment. Additionally, the expression of COX-2 in both groups is higher in the published graph compared to the repeat data (~5 and 10 fold difference for LPS and *T*. *cruzi*, respectively).

A member of the journal’s Editorial Board assessed these data and advised that the results of this repeat experiment support the article’s conclusions regarding the effect of *T*. *cruzi* infection on COX-2 expression in Raw cells, and that the method of density quantification is valid. They stated that results do not appear to be fully reproducible across replicas for COX-1, but did not express concern about this or the discrepancy between COX-2 expression in the published graph and repeat data.

Given the nature of the concerns, which cannot be fully resolved in the absence of the original underlying data, the *PLOS Neglected Tropical Diseases* Editors issue this Expression of Concern.

## Supporting information

S1 File Repeat of the experiment presented in Figs 2D and 2E in [1]. (PPTX)

S2 File Original images, quantitative data and method for repeat experiment presented in S1 File. A) Original western blot images. B) Western blots with ponceau staining and molecular weight markers for experiment in S1 File. C) Quantitative data. D) Protocol including quantification method. E) Plots for density quantification.
(ZIP)
