# Supplementary material for: Expression of Concern: Protective Role of Acetylsalicylic Acid in Experimental Trypanosoma cruzi Infection: Evidence of a 15-epi-Lipoxin A4-Mediated Effect
Source: PLoS Negl Trop Dis. 2024 Sep 5;18(9):e0012471. doi: 10.1371/journal.pntd.0012471 (PMC11376536; doi:10.1371/journal.pntd.0012471)
Supplement: S1 File — (PPTX) [file pntd.0012471.s001.pptx]

## Slide 1
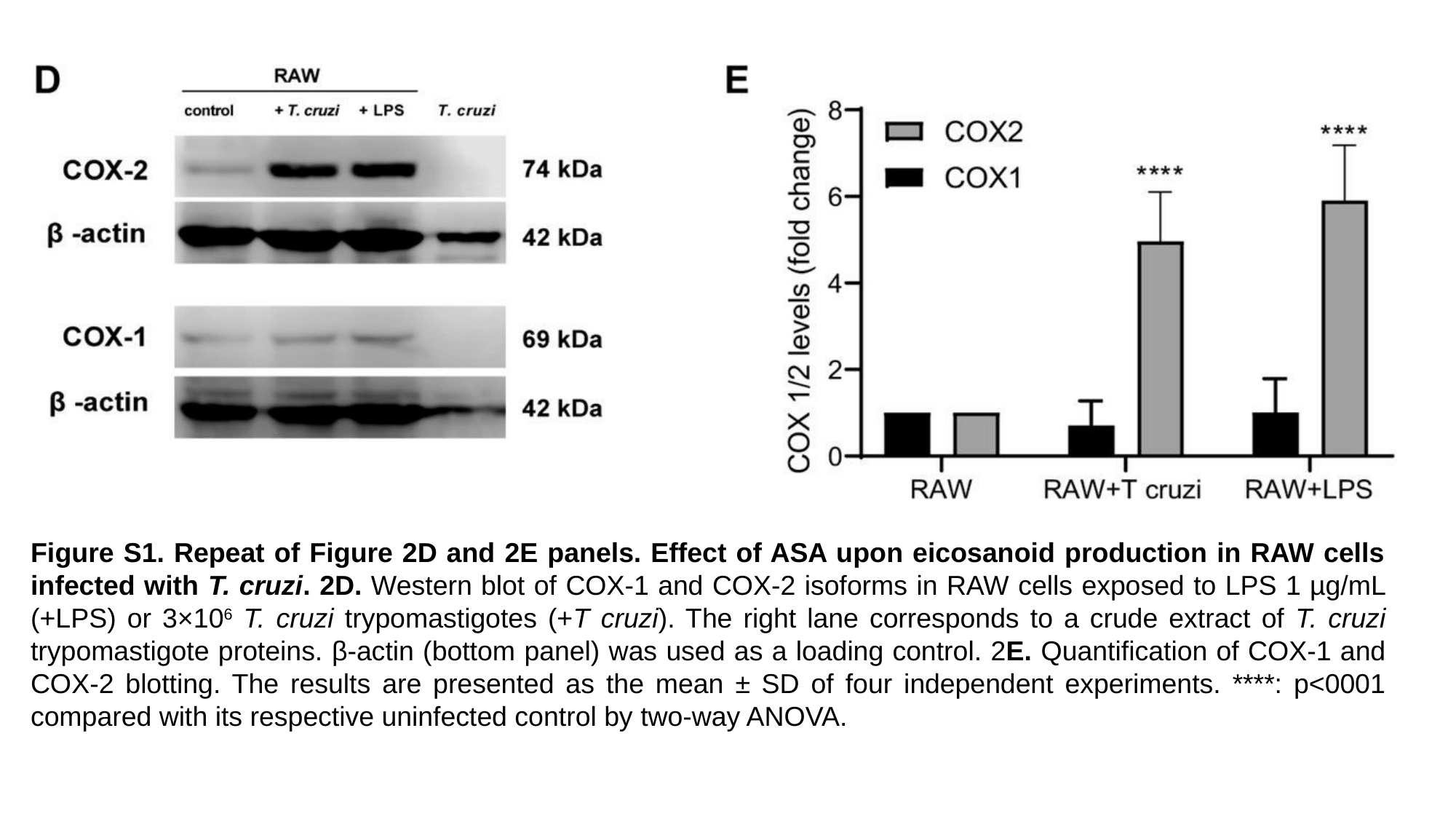

Figure S1. Repeat of Figure 2D and 2E panels. Effect of ASA upon eicosanoid production in RAW cells infected with T. cruzi. 2D. Western blot of COX-1 and COX-2 isoforms in RAW cells exposed to LPS 1 µg/mL (+LPS) or 3×10^6^ T. cruzi trypomastigotes (+T cruzi). The right lane corresponds to a crude extract of T. cruzi trypomastigote proteins. β-actin (bottom panel) was used as a loading control. 2E. Quantification of COX-1 and COX-2 blotting. The results are presented as the mean ± SD of four independent experiments. ****: p<0001 compared with its respective uninfected control by two-way ANOVA.
