# Supplementary material for: Expression of Concern: Protective Role of Acetylsalicylic Acid in Experimental Trypanosoma cruzi Infection: Evidence of a 15-epi-Lipoxin A4-Mediated Effect
Source: PLoS Negl Trop Dis. 2024 Sep 5;18(9):e0012471. doi: 10.1371/journal.pntd.0012471 (PMC11376536; doi:10.1371/journal.pntd.0012471)
Supplement: S2 File — A) Original western blot images. B) Western blots with ponceau staining and molecular weight markers for experiment in S1 File. C) Quantitative data. D) Protocol including quantification method. E) Plots for density quantification. (ZIP) [file pntd.0012471.s002.zip › S2 File/A. Original WBs for new figure 2D.pptx]

## Slide 1
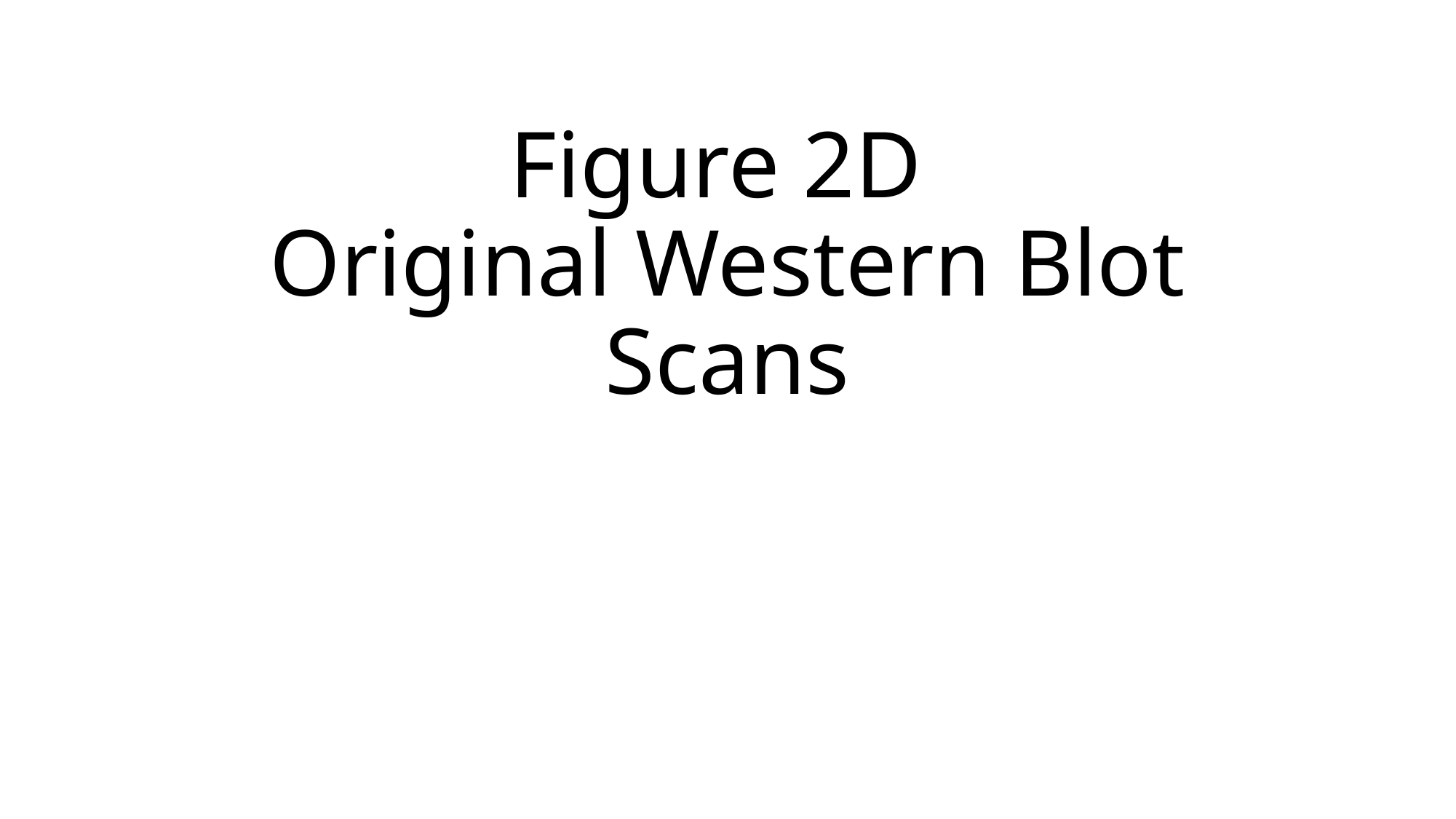

# Figure 2D Original Western Blot Scans

## Slide 2
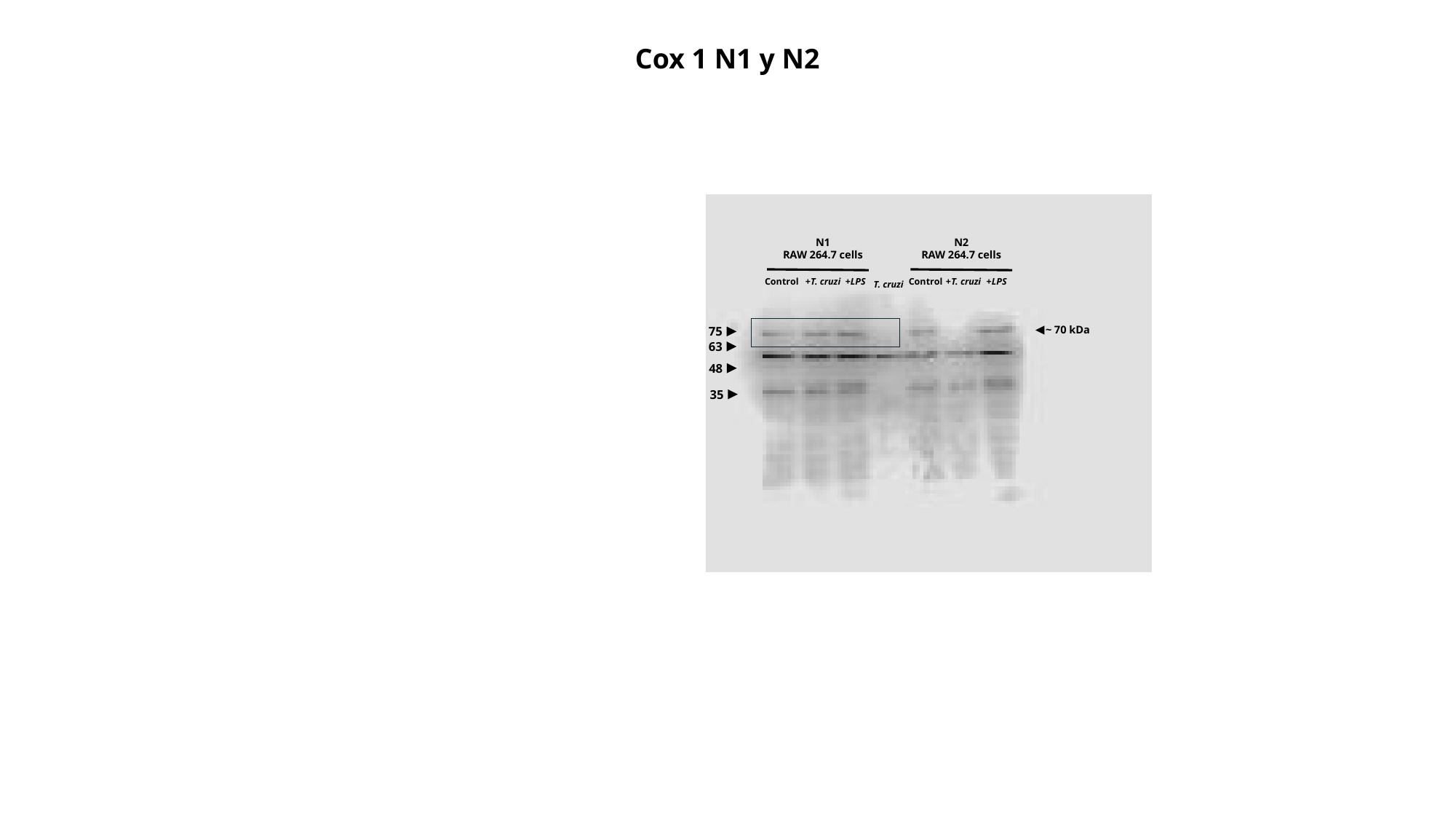

Cox 1 N1 y N2
N1
RAW 264.7 cells
N2
RAW 264.7 cells
N1
RAW 264.7 cells
N2
RAW 264.7 cells
Control
+T. cruzi
+LPS
Control
+T. cruzi
+LPS
T. cruzi
Control
+T. cruzi
+LPS
Control
+T. cruzi
+LPS
T. cruzi
~ 70 kDa
75
~ 70 kDa
75
63
63
48
48
35
35

## Slide 3
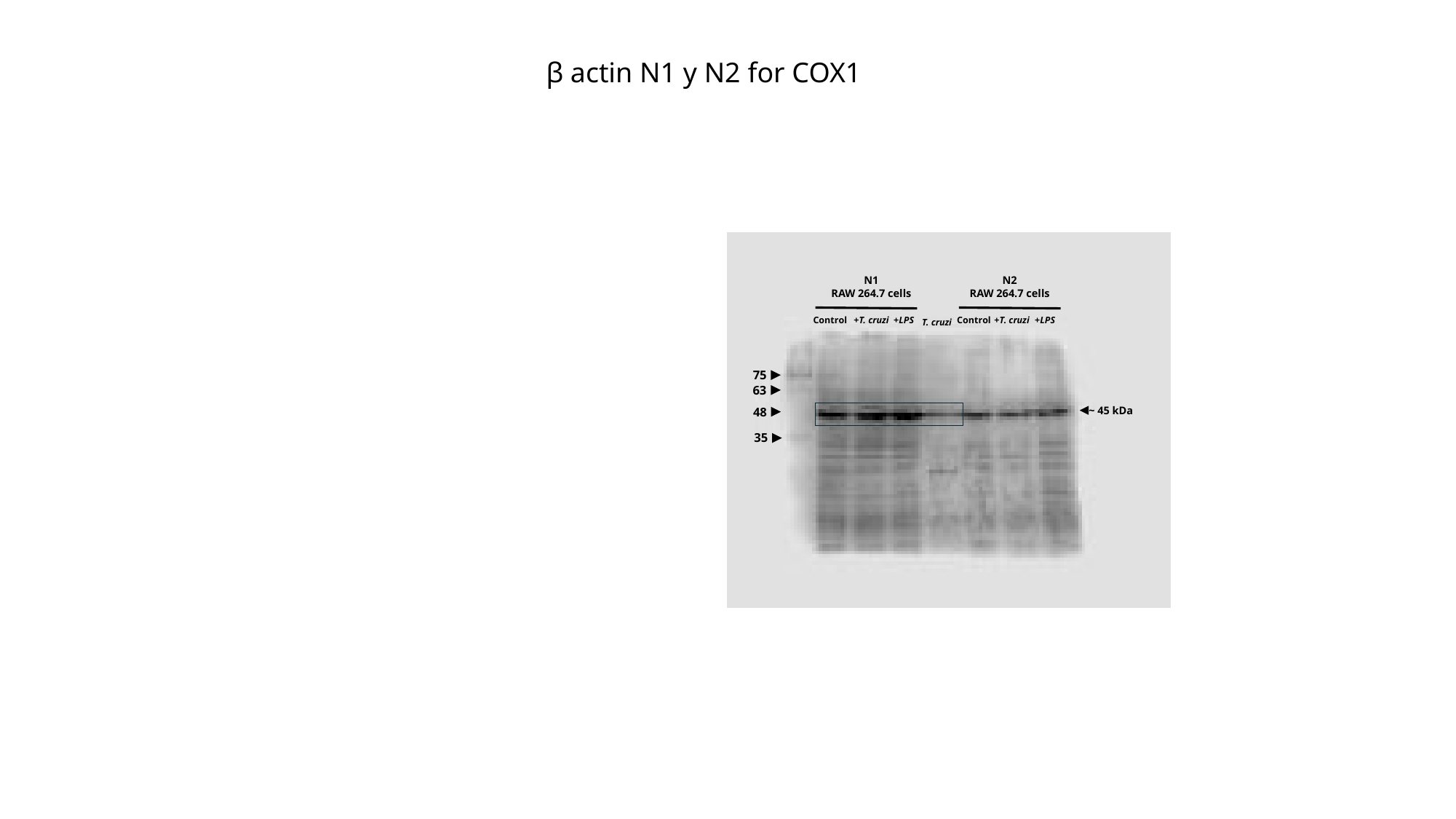

β actin N1 y N2 for COX1
N1
RAW 264.7 cells
N2
RAW 264.7 cells
N1
RAW 264.7 cells
N2
RAW 264.7 cells
Control
+T. cruzi
+LPS
Control
+T. cruzi
+LPS
Control
+T. cruzi
+LPS
Control
+T. cruzi
+LPS
T. cruzi
T. cruzi
75
75
63
63
~ 45 kDa
~ 45 kDa
48
48
35
35

## Slide 4
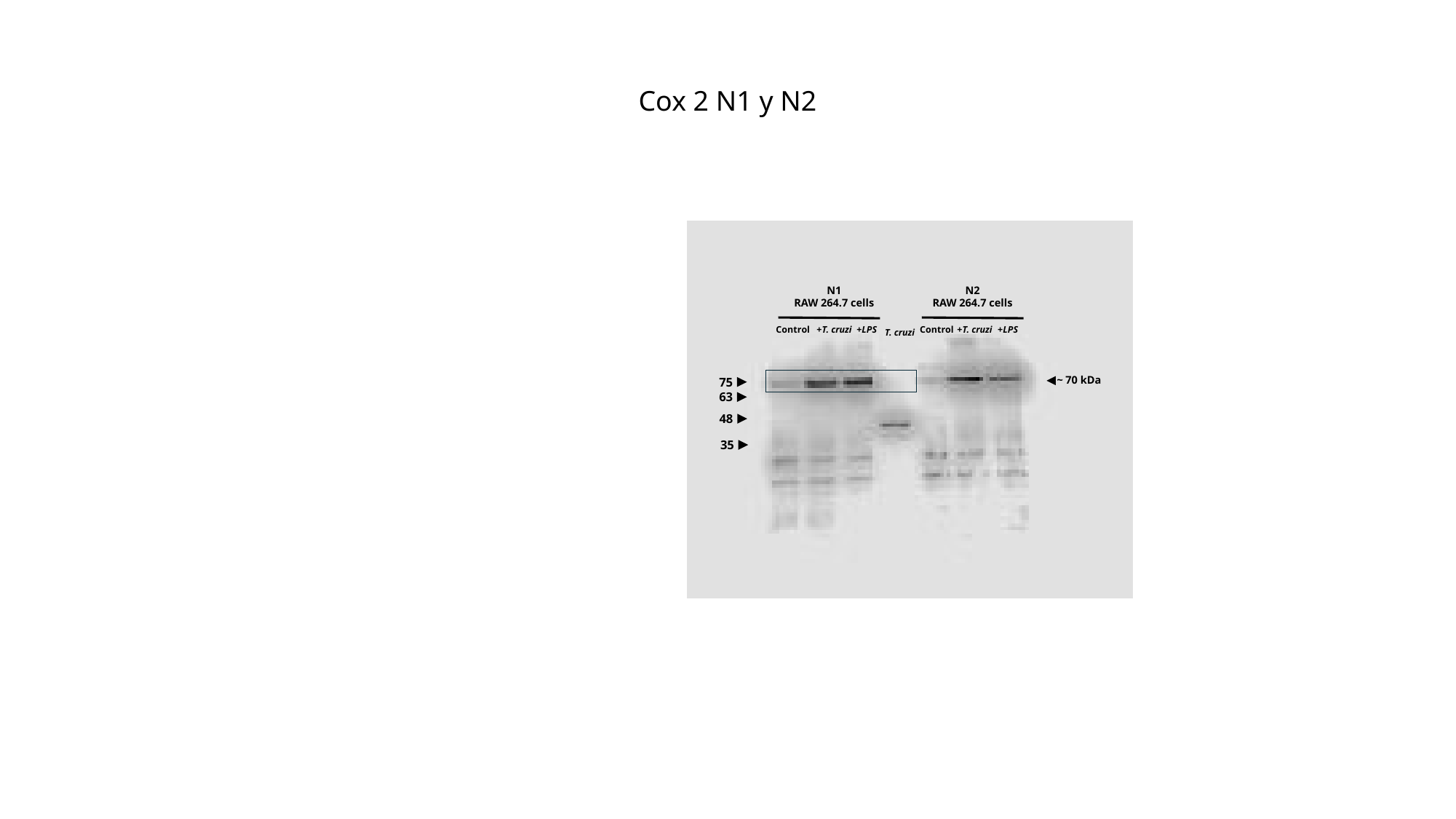

Cox 2 N1 y N2
N1
RAW 264.7 cells
N2
RAW 264.7 cells
N1
RAW 264.7 cells
N2
RAW 264.7 cells
Control
+T. cruzi
+LPS
Control
+T. cruzi
+LPS
T. cruzi
Control
+T. cruzi
+LPS
Control
+T. cruzi
+LPS
T. cruzi
~ 70 kDa
75
~ 70 kDa
75
63
63
48
48
35
35

## Slide 5
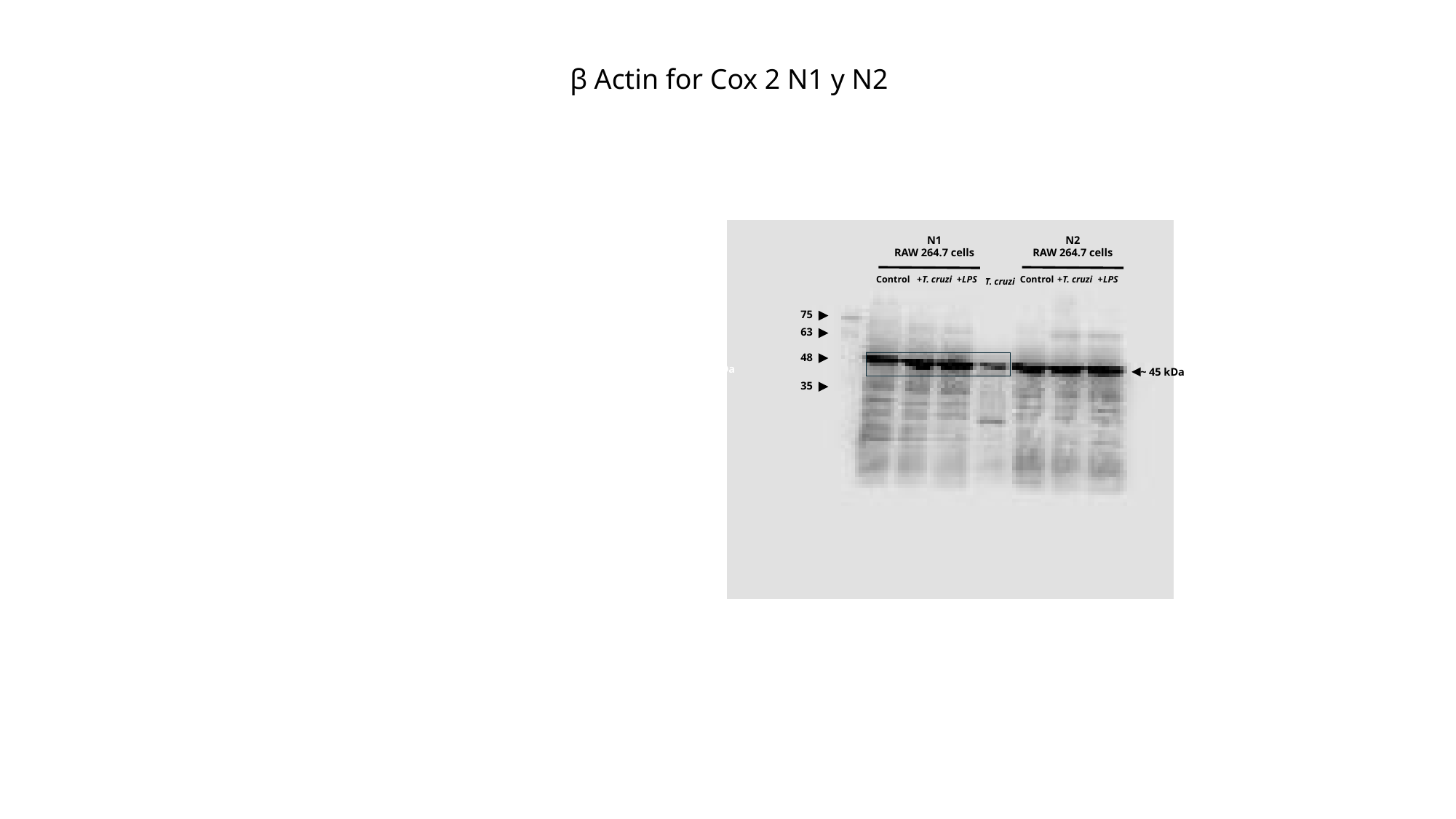

β Actin for Cox 2 N1 y N2
N1
RAW 264.7 cells
N2
RAW 264.7 cells
N1
RAW 264.7 cells
N2
RAW 264.7 cells
Control
+T. cruzi
+LPS
Control
+T. cruzi
+LPS
T. cruzi
Control
+T. cruzi
+LPS
Control
+T. cruzi
+LPS
T. cruzi
75
75
63
63
48
48
~ 45 kDa
~ 45 kDa
35
35

## Slide 6
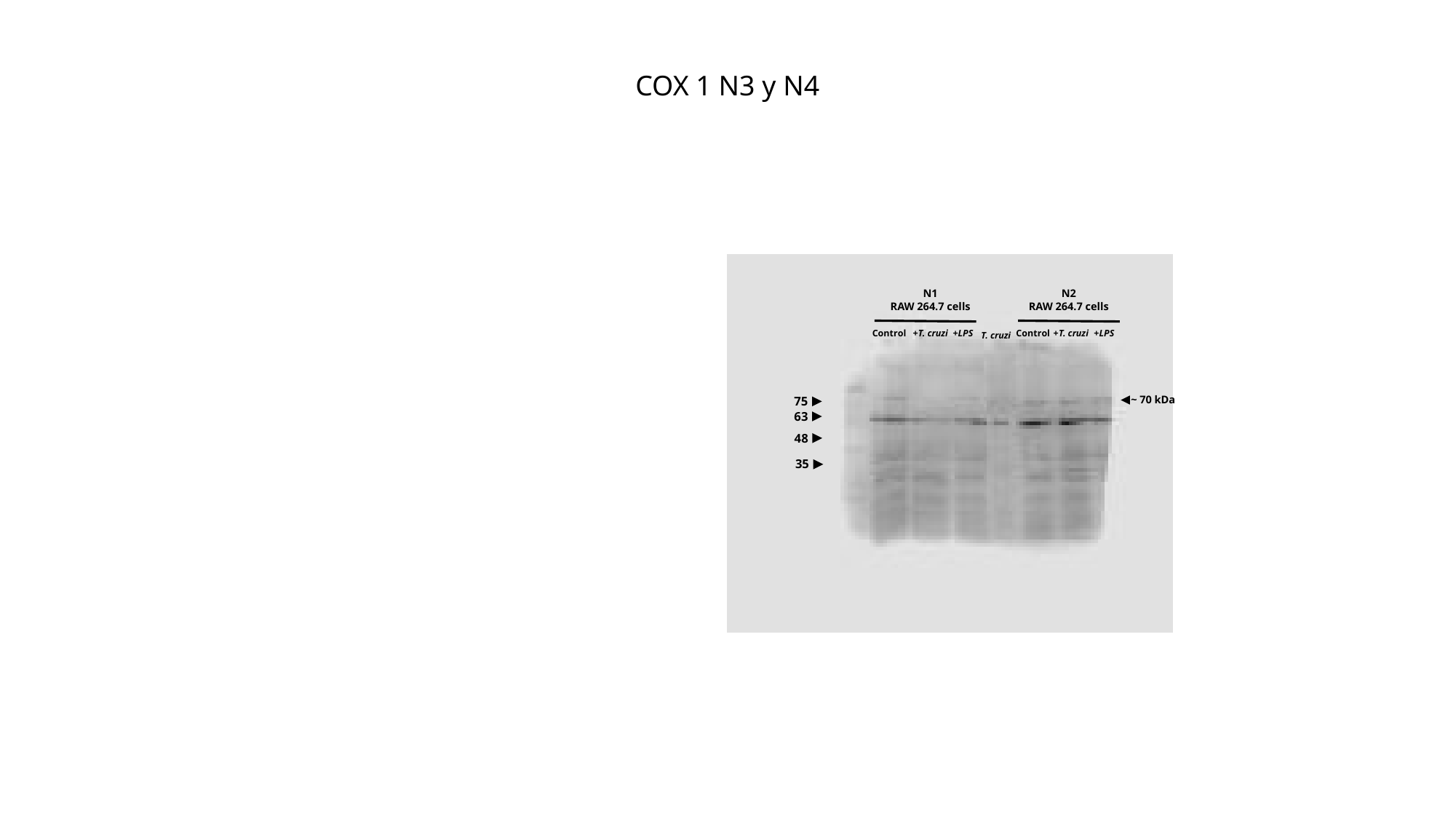

COX 1 N3 y N4
N1
RAW 264.7 cells
N2
RAW 264.7 cells
N1
RAW 264.7 cells
N2
RAW 264.7 cells
Control
+T. cruzi
+LPS
Control
+T. cruzi
+LPS
T. cruzi
Control
+T. cruzi
+LPS
Control
+T. cruzi
+LPS
T. cruzi
~ 70 kDa
75
~ 70 kDa
75
63
63
48
48
35
35

## Slide 7
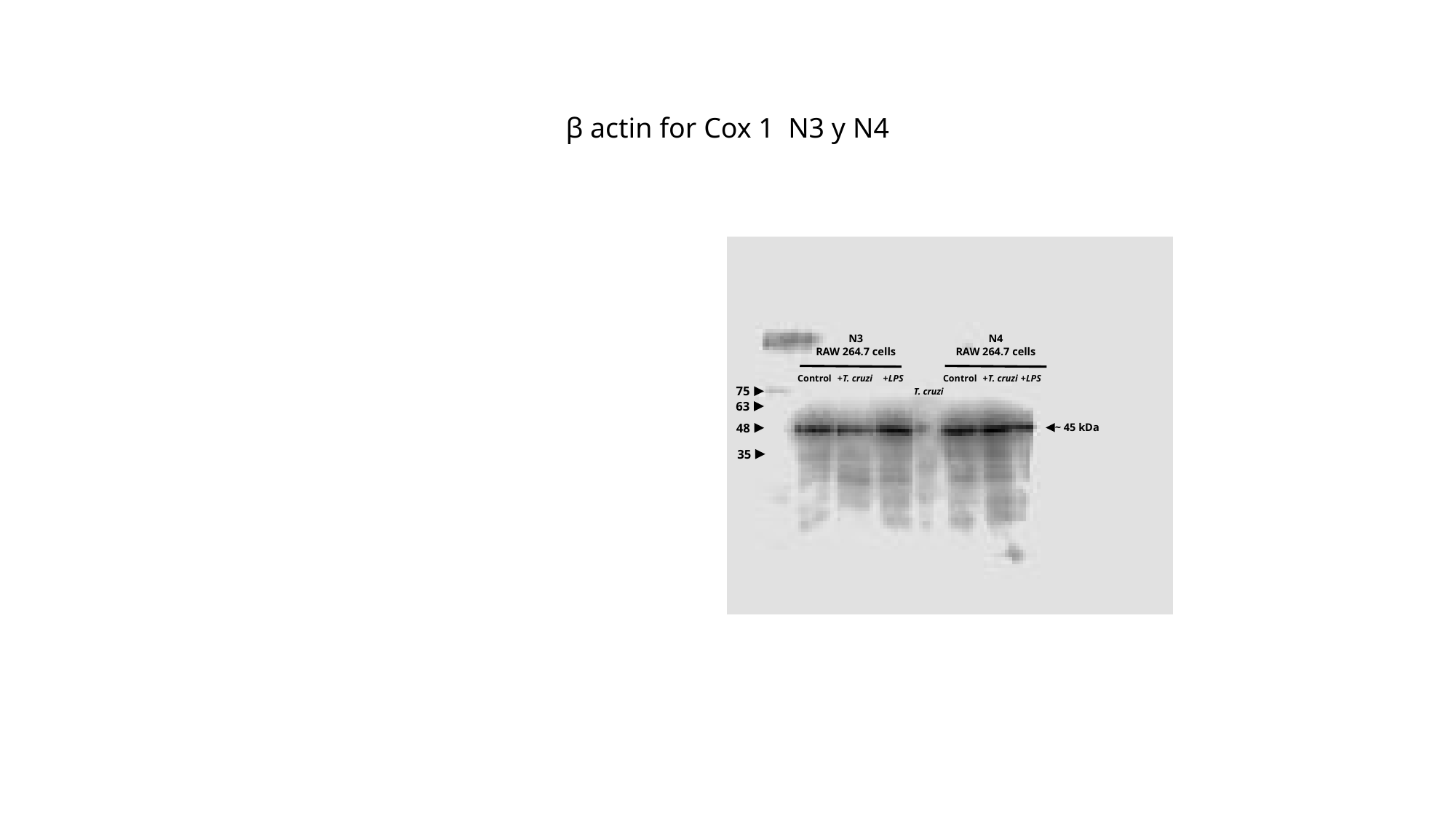

β actin for Cox 1 N3 y N4
N3
RAW 264.7 cells
N4
RAW 264.7 cells
N3
RAW 264.7 cells
N4
RAW 264.7 cells
Control
+T. cruzi
+LPS
Control
+T. cruzi
+LPS
Control
+T. cruzi
+LPS
Control
+T. cruzi
+LPS
75
75
T. cruzi
T. cruzi
63
63
48
48
~ 45 kDa
~ 45 kDa
35
35

## Slide 8
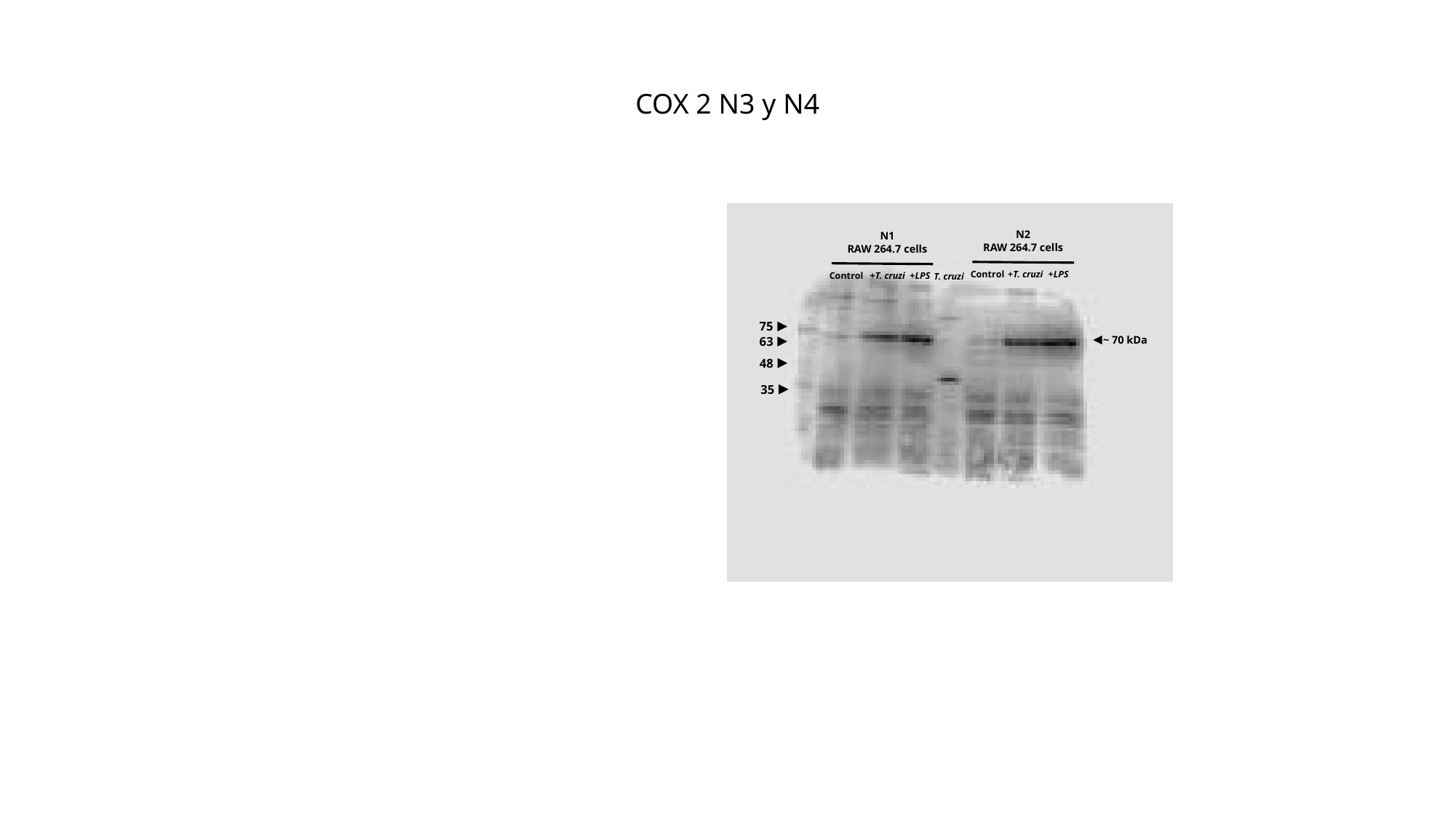

COX 2 N3 y N4
N1
RAW 264.7 cells
N2
RAW 264.7 cells
N2
RAW 264.7 cells
N1
RAW 264.7 cells
Control
+T. cruzi
+LPS
Control
+T. cruzi
+LPS
T. cruzi
Control
+T. cruzi
+LPS
Control
+T. cruzi
+LPS
T. cruzi
75
75
~ 70 kDa
63
~ 70 kDa
63
48
48
35
35

## Slide 9
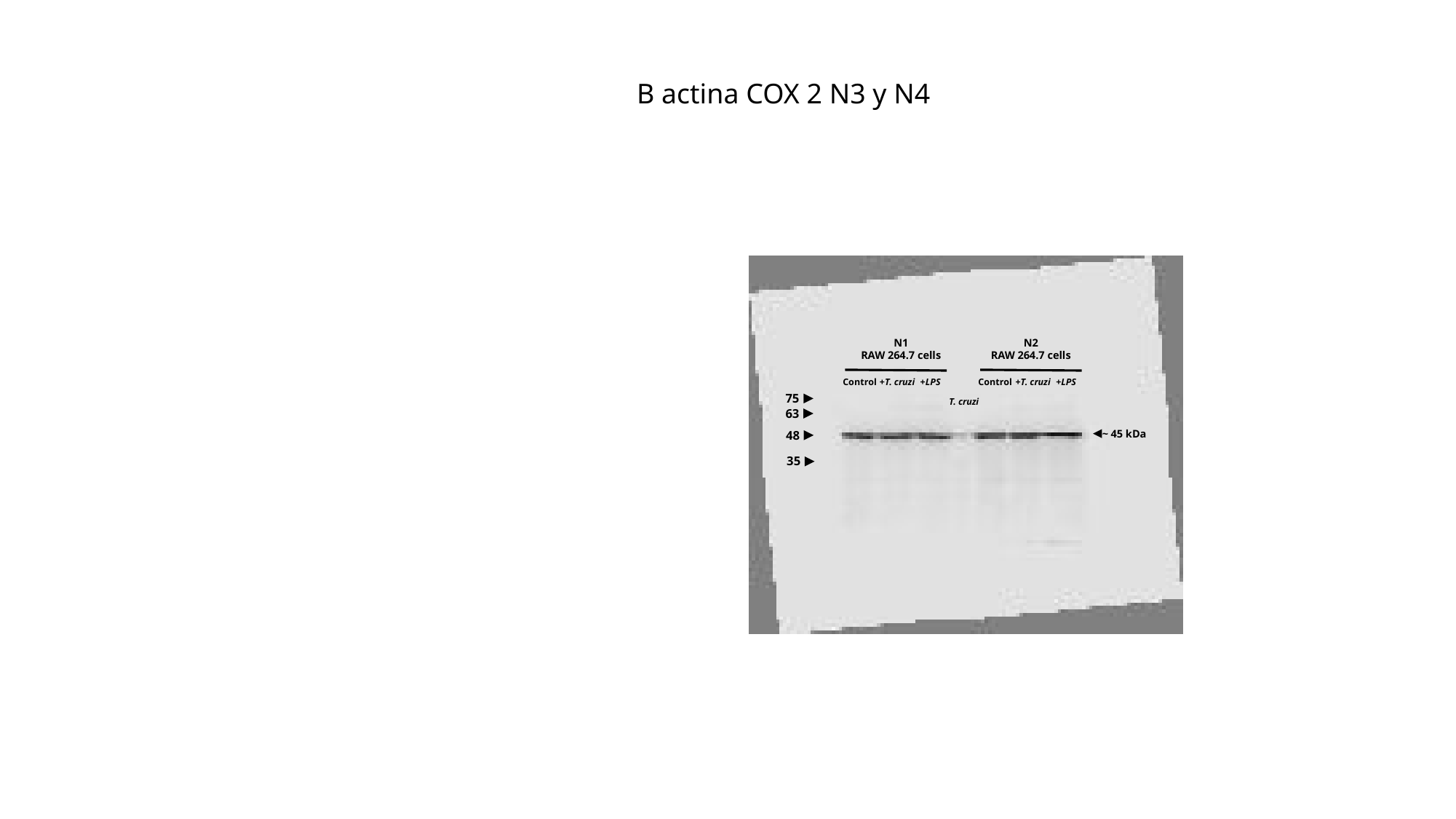

B actina COX 2 N3 y N4
N2
RAW 264.7 cells
N1
RAW 264.7 cells
N2
RAW 264.7 cells
N1
RAW 264.7 cells
Control
+T. cruzi
+LPS
Control
+T. cruzi
+LPS
Control
+T. cruzi
+LPS
Control
+T. cruzi
+LPS
75
75
T. cruzi
T. cruzi
63
63
~ 45 kDa
48
48
~ 45 kDa
35
35
