# Supplementary material for: Expression of Concern: Protective Role of Acetylsalicylic Acid in Experimental Trypanosoma cruzi Infection: Evidence of a 15-epi-Lipoxin A4-Mediated Effect
Source: PLoS Negl Trop Dis. 2024 Sep 5;18(9):e0012471. doi: 10.1371/journal.pntd.0012471 (PMC11376536; doi:10.1371/journal.pntd.0012471)
Supplement: S2 File — A) Original western blot images. B) Western blots with ponceau staining and molecular weight markers for experiment in S1 File. C) Quantitative data. D) Protocol including quantification method. E) Plots for density quantification. (ZIP) [file pntd.0012471.s002.zip › S2 File/D. Protocol for the Repeat Experiment of Figure 2D and 2E in S1 File.docx]

**Protocol for the Repeat Experiment of Figure 2D and 2E in S1 File**

RAW 264.7 cells (murine macrophages, ATCC number CRL-2922) were cultured at a density of 250,000 cells/cm^2^, in RPMI 1640 medium, supplemented with 5% fetal bovine serum, in humidified air with 5% CO_2_, at 37ºC. RAW cells were infected with *T. cruzi* trypomastigotes (Dm28c strain) at a 3:1 ratio (trypomastigote:RAW cell). Trypomastigotes were allowed to infect cells for 24 hours. Then, cells were washed twice with sterile PBS (pH 7.4) to extract extracellular trypomastigotes.

For protein isolation, RAW cells were washed with PBS, scraped with RIPA buffer (Sigma-Aldrich), and lysed by sonication for 30 seconds in an ultrasonic sonicator bath (Biobase, Shandong, China). All samples were homogenized in lysis buffer at pH 8, containing Tris 0.01 μM, SDS 1%, and protease inhibitor cocktail (Complete Mini EDTA-free, Roche, Indianapolis, IN, USA). Total protein was quantified using the bicinchoninic acid method, using the BCA Pierce kit (Pierce Biotechnology, Waltham, MA, USA), following manufacturer instructions.

For electrophoresis, extracted proteins were mixed with loading buffer (10% SDS, 50% glycerol, 0.5 M Tris, 0.1% bromophenol blue, and 1 M dithiothreitol, pH 6.8), and 40 μg of total protein was loaded into 8% polyacrylamide gels. After, proteins were electron transferred to nitrocellulose membranes in a Trans-blot semi-dry system (Bio-Rad, Hercules, CA, USA) and were blocked for 2 hr with BSA (3% in TBST 0.05%). After three washes with TBST, membranes were incubated overnight at 4ºC with primary polyclonal antibodies against COX-1 (**AB109025, Abcam, Cambridge, UK, dilution 1:1000 v/v, instead of AB59964** **used in the original version of the paper**), COX-2 (AB52237, Abcam, Cambridge, UK, dilution 1:1000 v/v), diluted in PBST-BSA 3% v/v. Membranes were washed with TBST 0,05%, and incubated with a secondary antibody (Anti-rabbit IgG, HRP-linked Antibody #7074, Cell Signalling Technology, Danvers, MA, USA, dilution 1:200 v/v ) for 1 hour. Afterward, membranes were washed with TBST and developed through chemiluminescence using the Immobilon Forte Western HRP substrate (Merck Millipore, Burlington, MA, USA). After that, membranes were incubated on stripping solution (glycine 200 mM, 3.467 mM SDS, 1% v/v Tween20, pH 2.2) and 50) at RT for 10 minutes. Membranes were washed in TBST, blocked again, and incubated with a primary antibody against β-actin (AB3280, Abcam, Cambridge, UK) overnight at 4°C. We used Anti-mouse IgG, HRP-linked Antibody #7076 (Cell Signaling Technology, Danvers, MA, USA.) as the secondary antibody.

**Image acquisition was changed from film to digital scanning**, using a LI-COR C-Digit blot scanner (LI-COR, Lincoln, NE, USA) of the chemiluminescence signals of the secondary antibodies. Band densitometry analysis of digital images was performed using the free software ImageJ (Ver 1.54f).

**We performed a new densitometric analysis, generating the same baseline for all bands so that the area under the curve was calculated using the same baseline for all spots using the following procedure**:

After identifying the bands according to molecular weight (see image showing the complete gel), quantification was performed using Image J Version 1.54f.

For all evaluations, treated cells with their respective control were considered for each experimental replicate to allow a proper comparison between controls and treatments.

To address the concern about the differences in published results, we considered that that is due to an overestimation of the data. Thus, a new approach was performed, redrawing a new common baseline and “normalizing” the background.

A representation of the quantitation method is shown in the following figure:


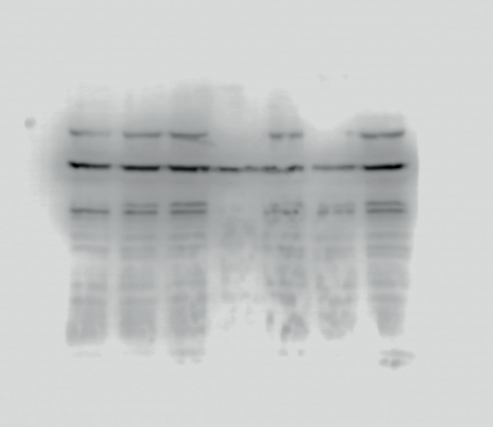


Common baseline. “normalized” background


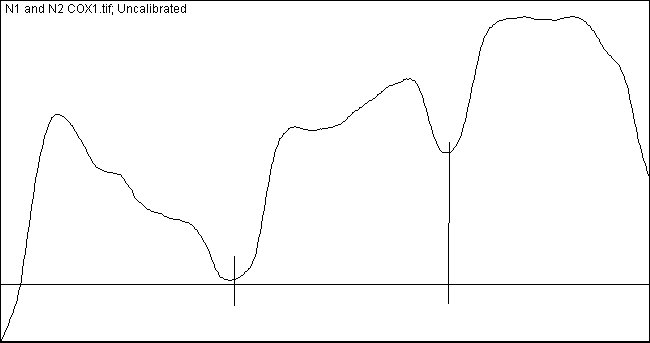


We used ImageJ Software Version 1.54f. After loading the image, the three lanes corresponding to each assay were selected. In the example image below, the selection of replicate 1 (N1) is shown in the gel with which we revealed COX lines (COX 1 and COX2).


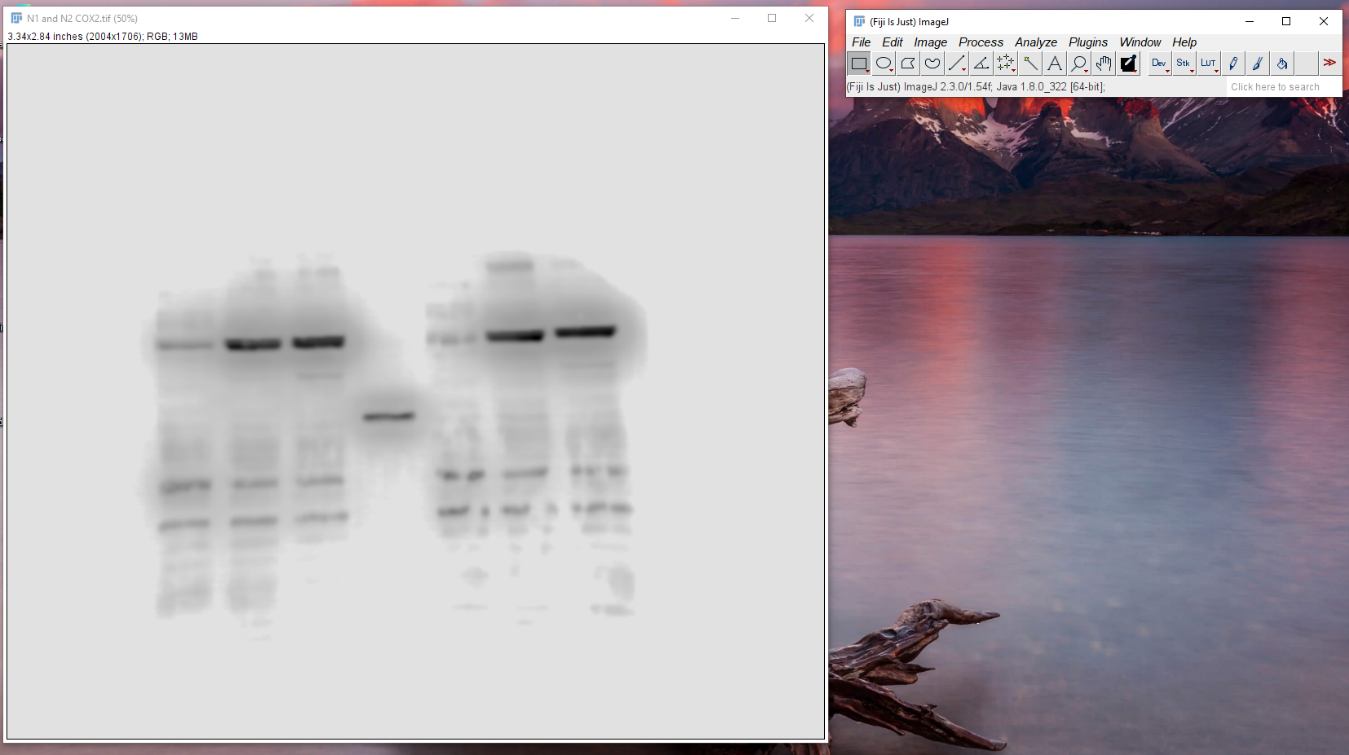


We selected the three lanes of the same replicate by drawing a rectangle containing these lanes. Then, we use the "analyze" tool and follow the sequence Analyze 🡪 Gels 🡪 Select first lane.


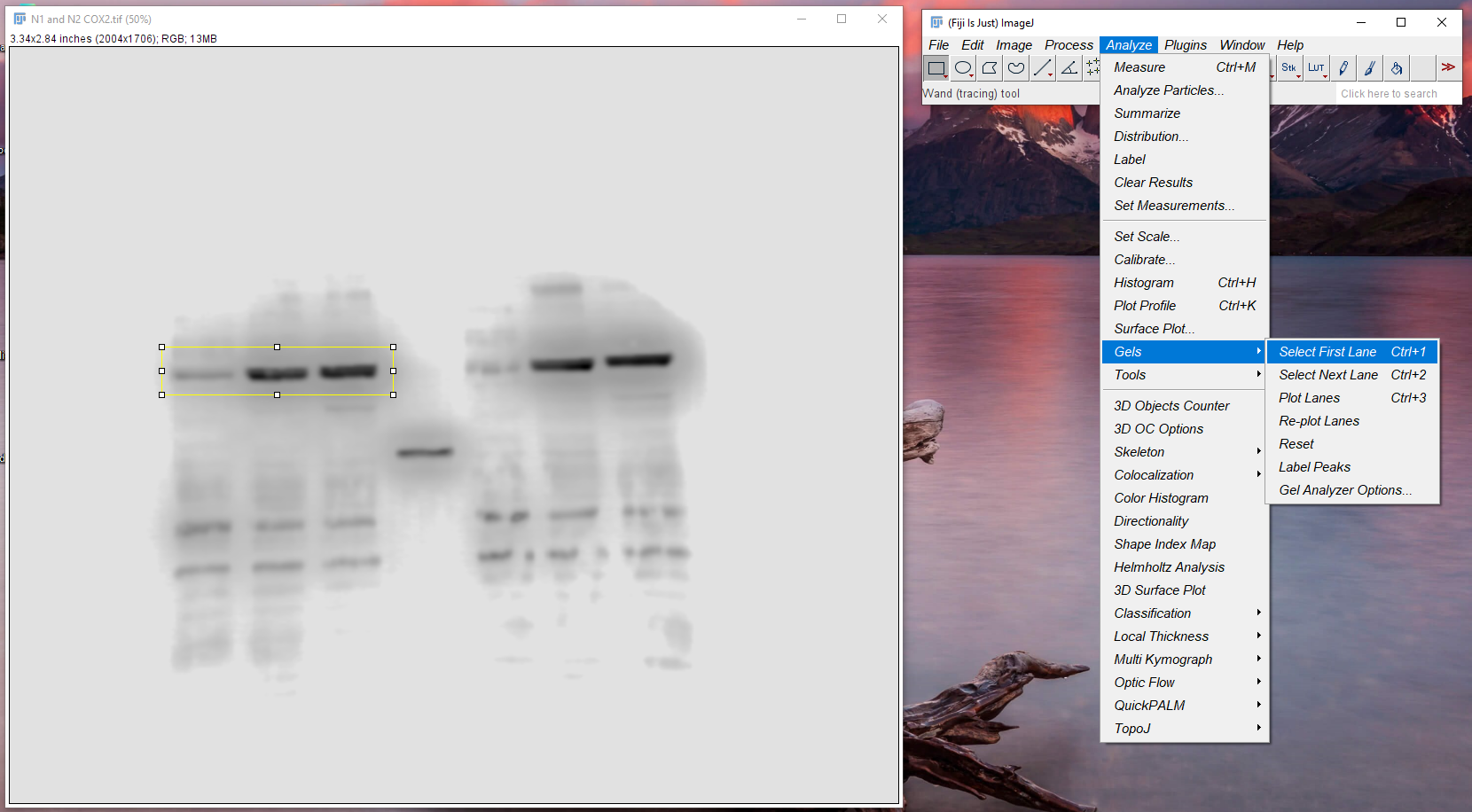


After selecting the first lane, we proceed with the sequence: Analyze 🡪 Gels 🡪 Plot lanes


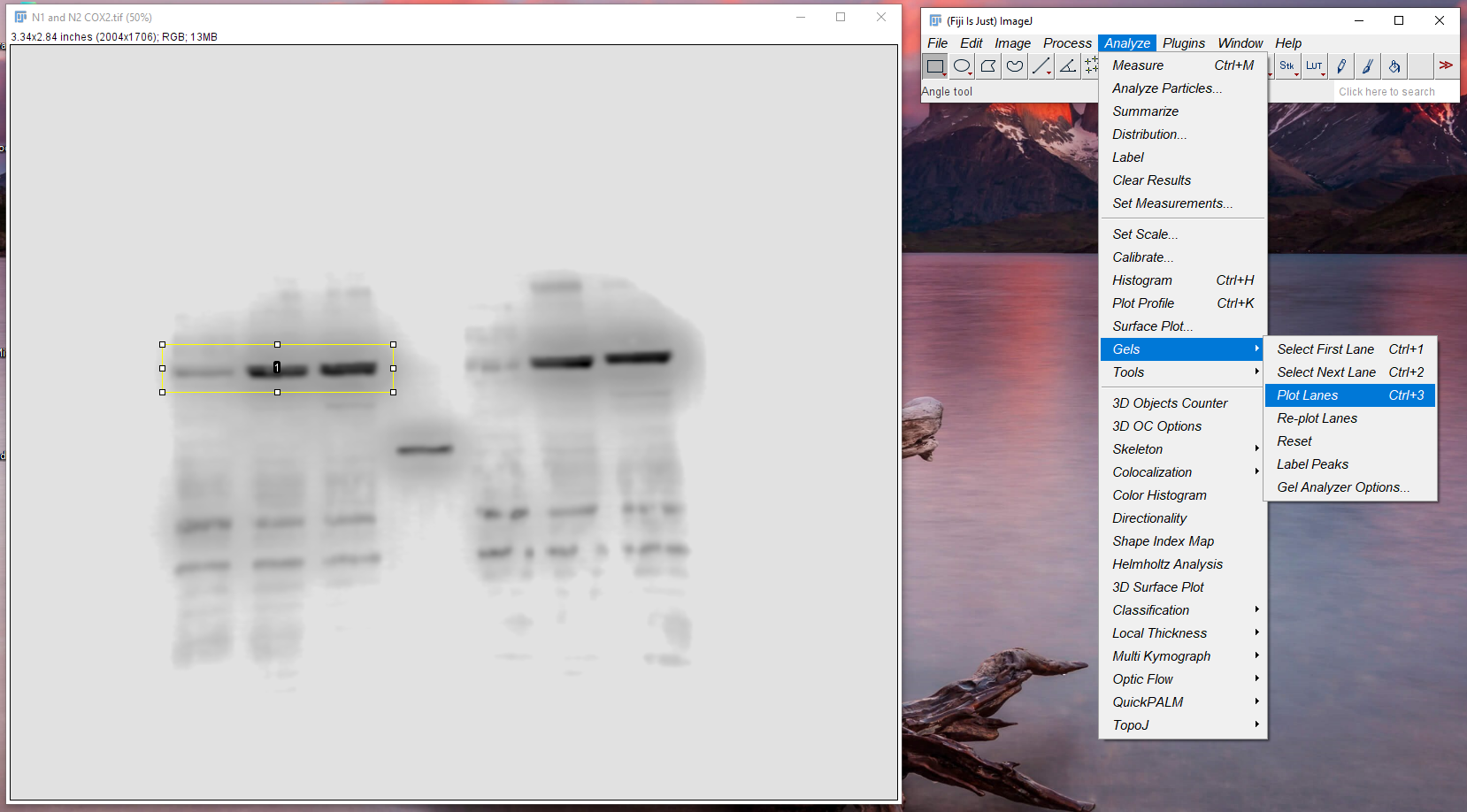


The previous sequence allowed us to obtain a plot showing the difference in pixel densities between lanes.


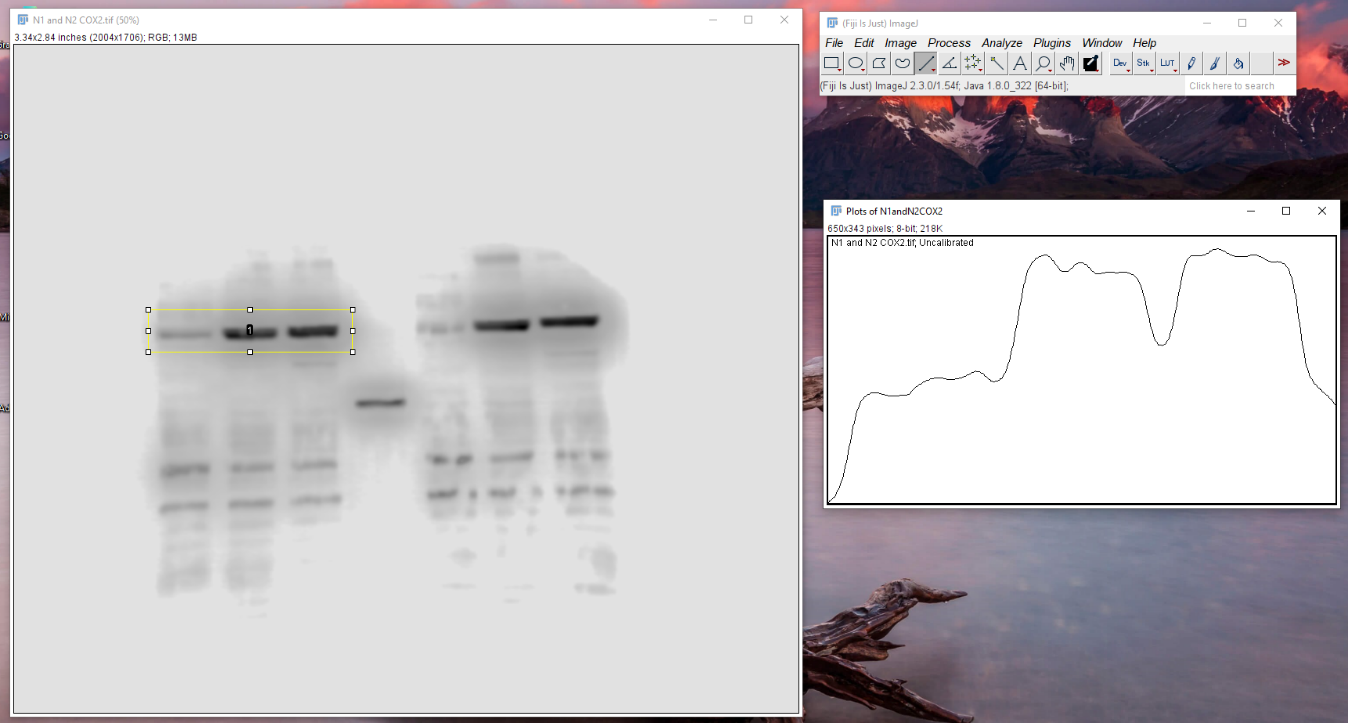


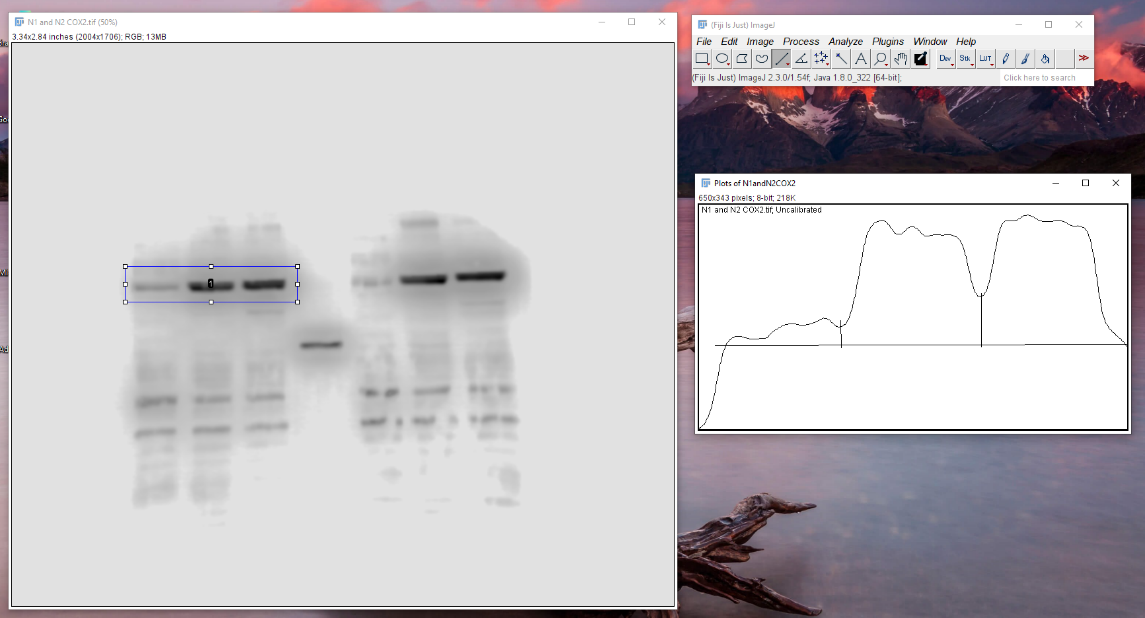
To standardize the background of the analysis, we drew a horizontal line at a height that we considered reasonable to clean the background of all lanes uniformly. Additionally, we drew horizontal lines to separate each lane.


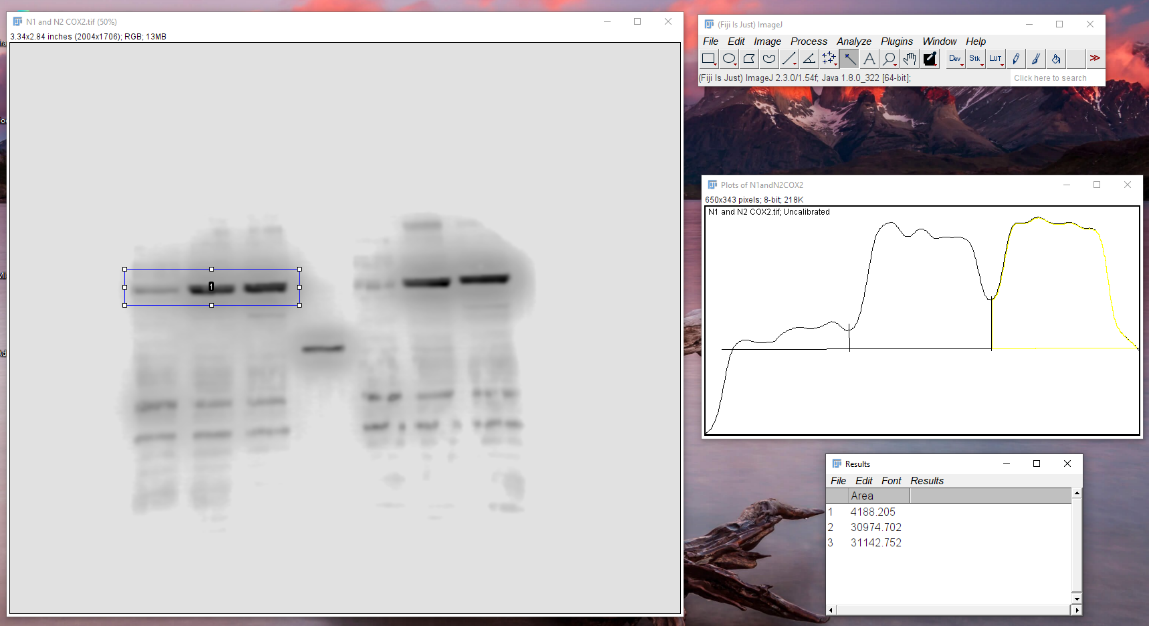
Finally, using the "wand (tracing)" tool, we selected each generated area, which provided us with a pixel quantification of each peak.

This procedure was used for each replicate, quantifying COX1 and COX2 and the loading control (β-actin). All plots resulting from the analysis of the bands by ImageJ are shown below (N corresponds to the replicate number):

**COX-1**

**β-actin**


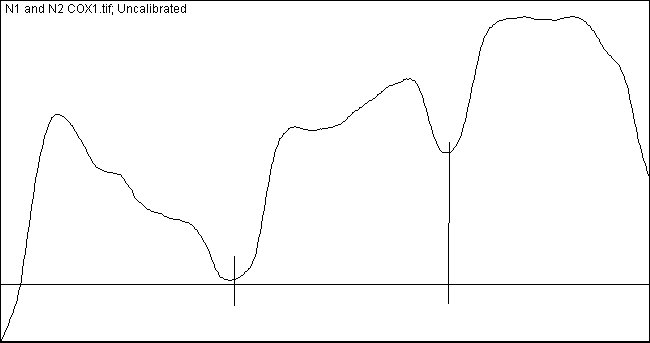

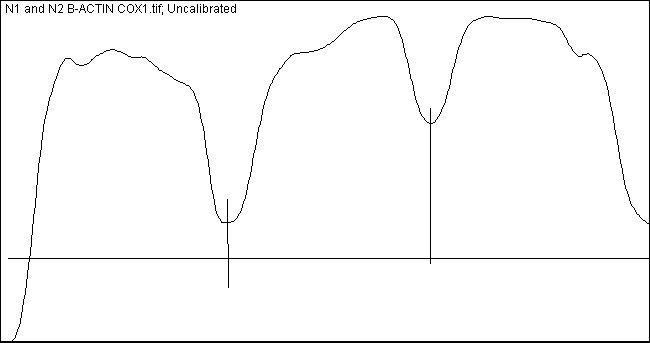

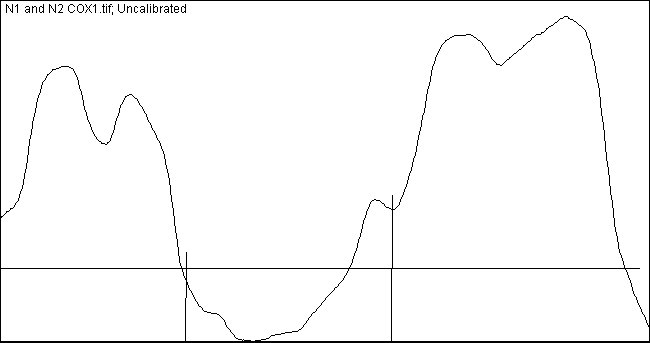

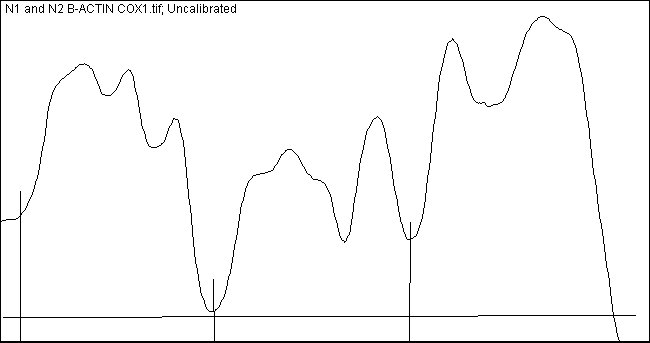

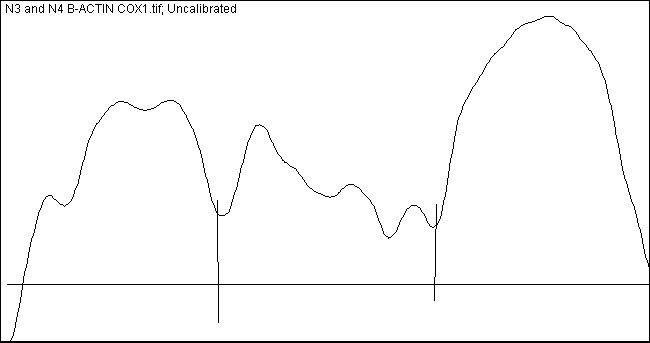

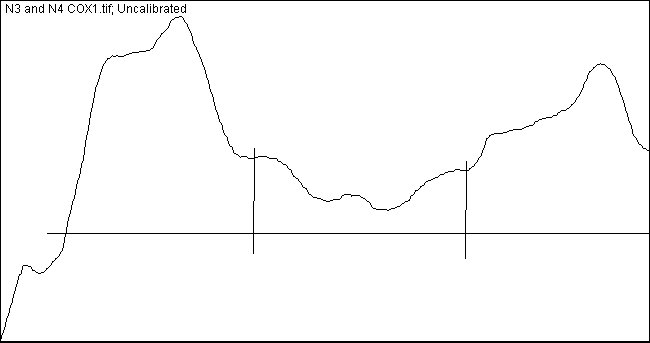

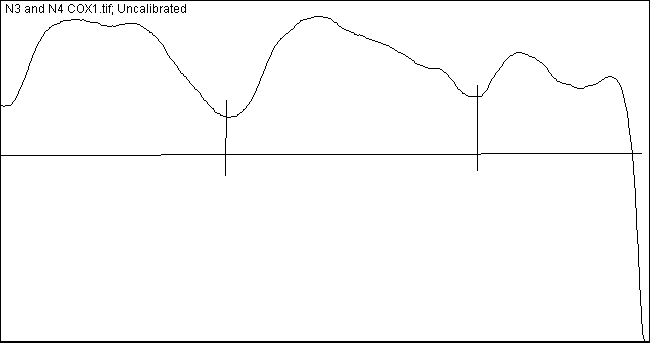

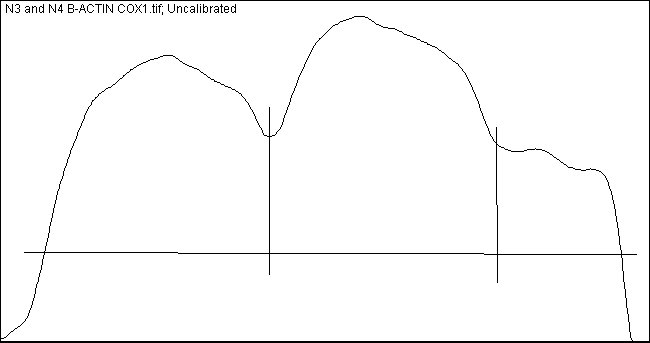


**N1**

**N2**

**N3**

**N4**

**control**

**T. cruzi**

**LPS**

**control**

**T. cruzi**

**LPS**

**COX-2**

**b-actina**





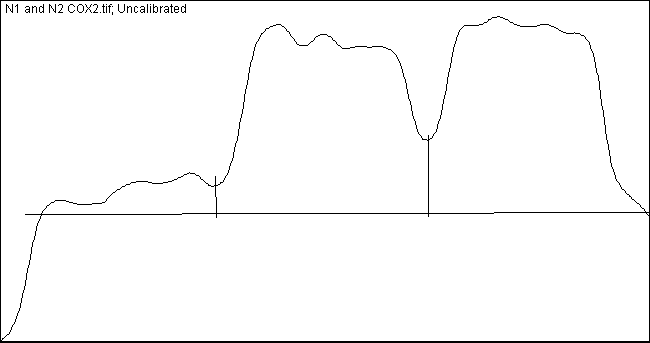

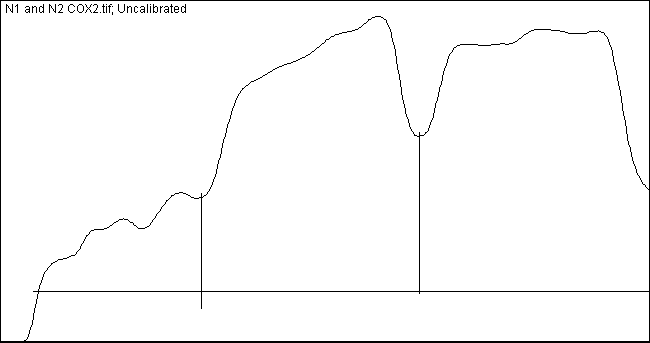

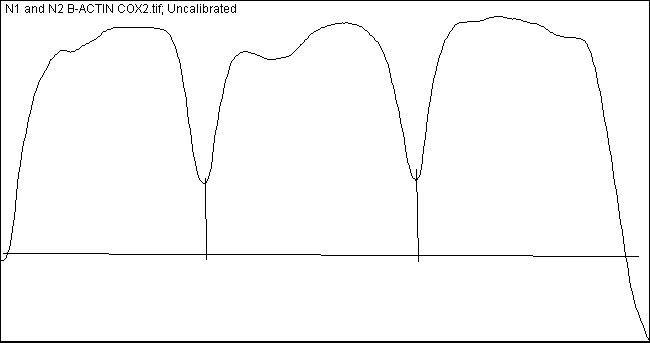

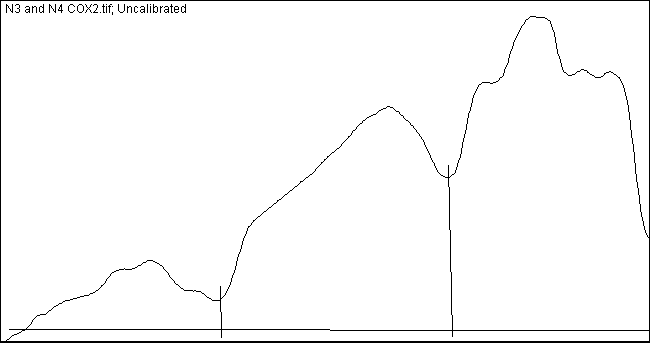

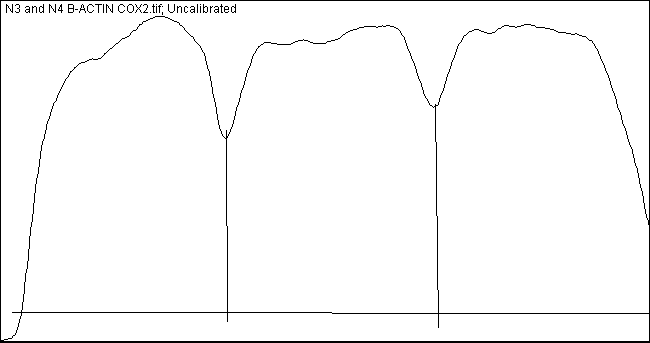

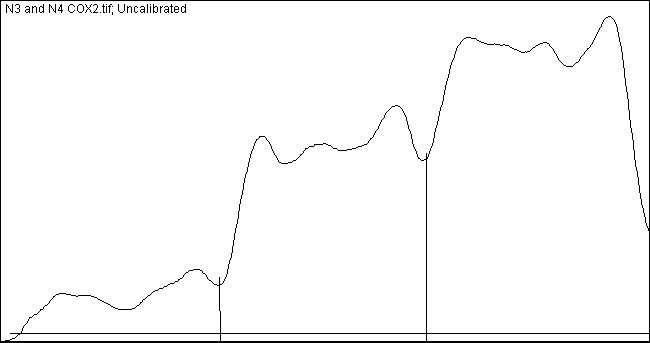

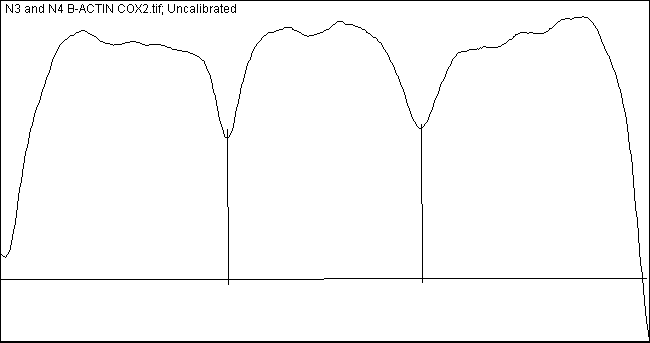


**N1**

**N2**

**N3**

**N4**

**control**

**T. cruzi**

**LPS**

**control**

**T. cruzi**

**LPS**

The pixel values of COX (1 or 2) were divided by their respective loading control and then normalized by the control of each replicate, using the following table (the Excel file is attached):

|  |  |  | COX | B-ACTIN | COX/B-ACTIN | NORM |
| --- | --- | --- | --- | --- | --- | --- |
| **N1** | **COX2** | **RAW** | 4188 | 32691 | 0.13 | **1.00** |
|  |  | **RAW + T. cruzi** | 30975 | 37009 | 0.84 | **6.53** |
|  |  | **RAW + LPS** | 31143 | 39890 | 0.78 | **6.09** |
|  | **COX1** | **RAW** | 19091 | 32727 | 0.58 | **1.00** |
|  |  | **RAW + T. cruzi** | 31415 | 37387 | 0.84 | **1.44** |
|  |  | **RAW + LPS** | 46159 | 41606 | 1.11 | **1.90** |
|  |  |  |  |  |  |  |
|  |  |  | COX | B-ACTIN | COX/B-ACTIN | NORM |
| **N2** | **COX2** | **RAW** | 10314 | 36163 | 0.29 | **1.00** |
|  |  | **RAW + T. cruzi** | 47447 | 40402 | 1.17 | **4.12** |
|  |  | **RAW + LPS** | 52568 | 41525 | 1.27 | **4.44** |
|  | **COX1** | **RAW** | 24985 | 35235 | 0.71 | **1.00** |
|  |  | **RAW + T. cruzi** | 2118 | 24682 | 0.09 | **0.12** |
|  |  | **RAW + LPS** | 44354 | 44157 | 1.00 | **1.42** |
|  |  |  |  |  |  |  |
|  |  |  | COX | B-ACTIN | COX/B-ACTIN | NORM |
| **N3** | **COX2** | **RAW** | 7964 | 49150 | 0.16 | **1.00** |
|  |  | **RAW + T. cruzi** | 36648 | 54950 | 0.67 | **4.12** |
|  |  | **RAW + LPS** | 49044 | 54010 | 0.91 | **5.60** |
|  | **COX1** | **RAW** | 27471 | 26249 | 1.0 | **1.00** |
|  |  | **RAW + T. cruzi** | 9956 | 20832 | 0.5 | **0.46** |
|  |  | **RAW + LPS** | 21391 | 41992 | 0.5 | **0.49** |
|  |  |  |  |  |  |  |
|  |  |  | COX | B-ACTIN | COX/B-ACTIN | NORM |
| **N4** | **COX2** | **RAW** | 7564 | 46103 | 0.16 | **1.00** |
|  |  | **RAW + T. cruzi** | 36822 | 44165 | 0.83 | **5.08** |
|  |  | **RAW + LPS** | 59491 | 48304 | 1.23 | **7.51** |
|  | **COX1** | **RAW** | 23101 | 34745 | 0.7 | **1.00** |
|  |  | **RAW + T. cruzi** | 24581 | 44577 | 0.6 | **0.83** |
|  |  | **RAW + LPS** | 11771 | 78554 | 0.1 | **0.23** |

After quantification, the values were as follows:

|  | **COX1** | | | **COX2** | | |
| --- | --- | --- | --- | --- | --- | --- |
|  | **MEAN** | **SD** | **N** | **MEAN** | **SD** | **N** |
| **RAW** | *1.00* | *0.00* | *4.00* | *1.00* | *0.00* | *4.00* |
| **RAW+T cruzi** | *0.71* | *0.57* | *4.00* | *4.96* | *1.14* | *4.00* |
| **RAW+LPS** | *1.01* | *0.78* | *4.00* | *5.91* | *1.27* | *4.00* |

Note that the controls (RAW) do not have a standard deviation because they were used to normalize each trial; therefore, they were always assigned the value of 1.
