# Supplementary material for: Expression of Concern: Protective Role of Acetylsalicylic Acid in Experimental Trypanosoma cruzi Infection: Evidence of a 15-epi-Lipoxin A4-Mediated Effect
Source: PLoS Negl Trop Dis. 2024 Sep 5;18(9):e0012471. doi: 10.1371/journal.pntd.0012471 (PMC11376536; doi:10.1371/journal.pntd.0012471)
Supplement: S2 File — A) Original western blot images. B) Western blots with ponceau staining and molecular weight markers for experiment in S1 File. C) Quantitative data. D) Protocol including quantification method. E) Plots for density quantification. (ZIP) [file pntd.0012471.s002.zip › S2 File/E. Plot for Densit Quantification COX1 and COX2.pptx]

## Slide 1
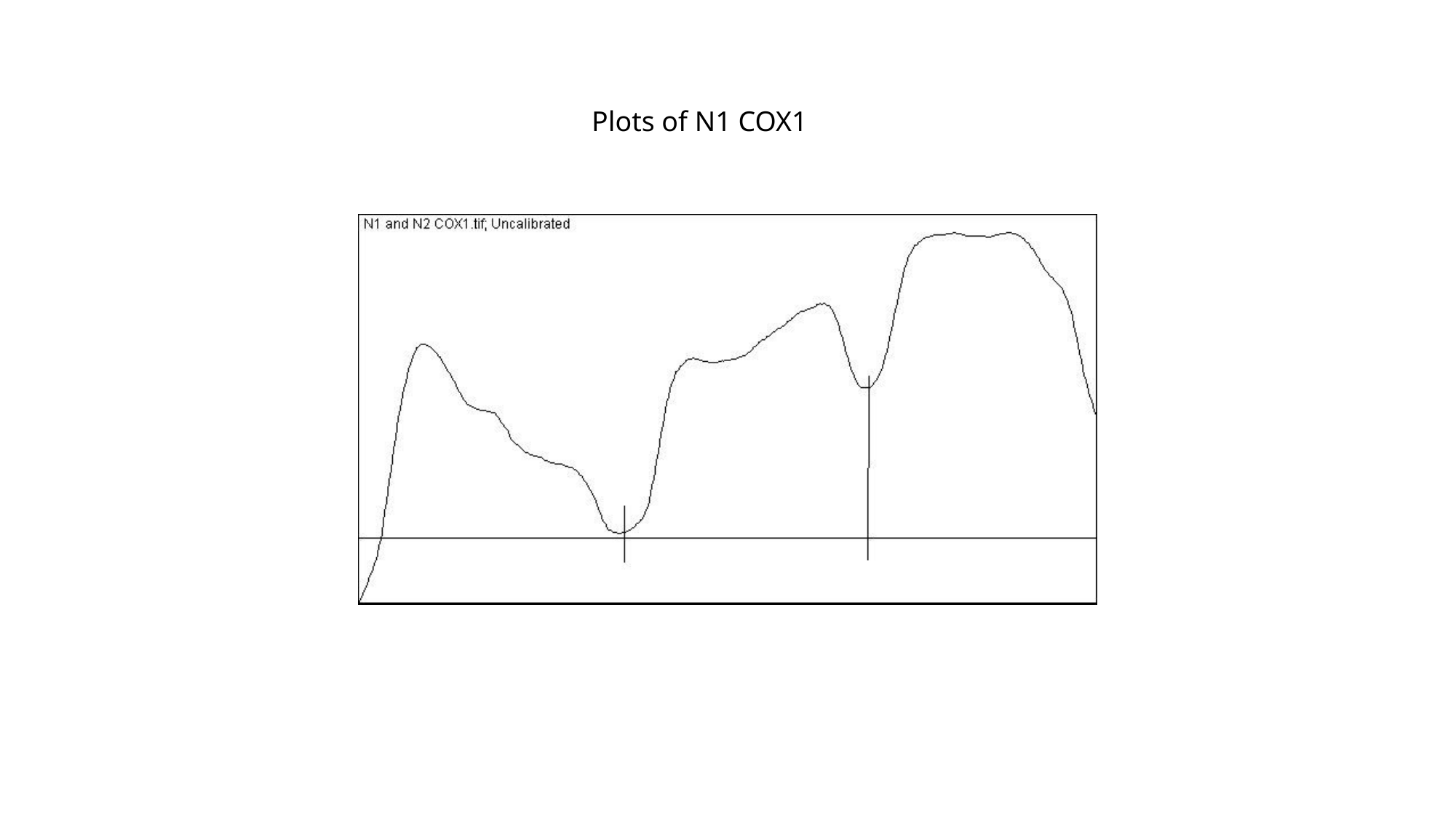

Plots of N1 COX1

## Slide 2
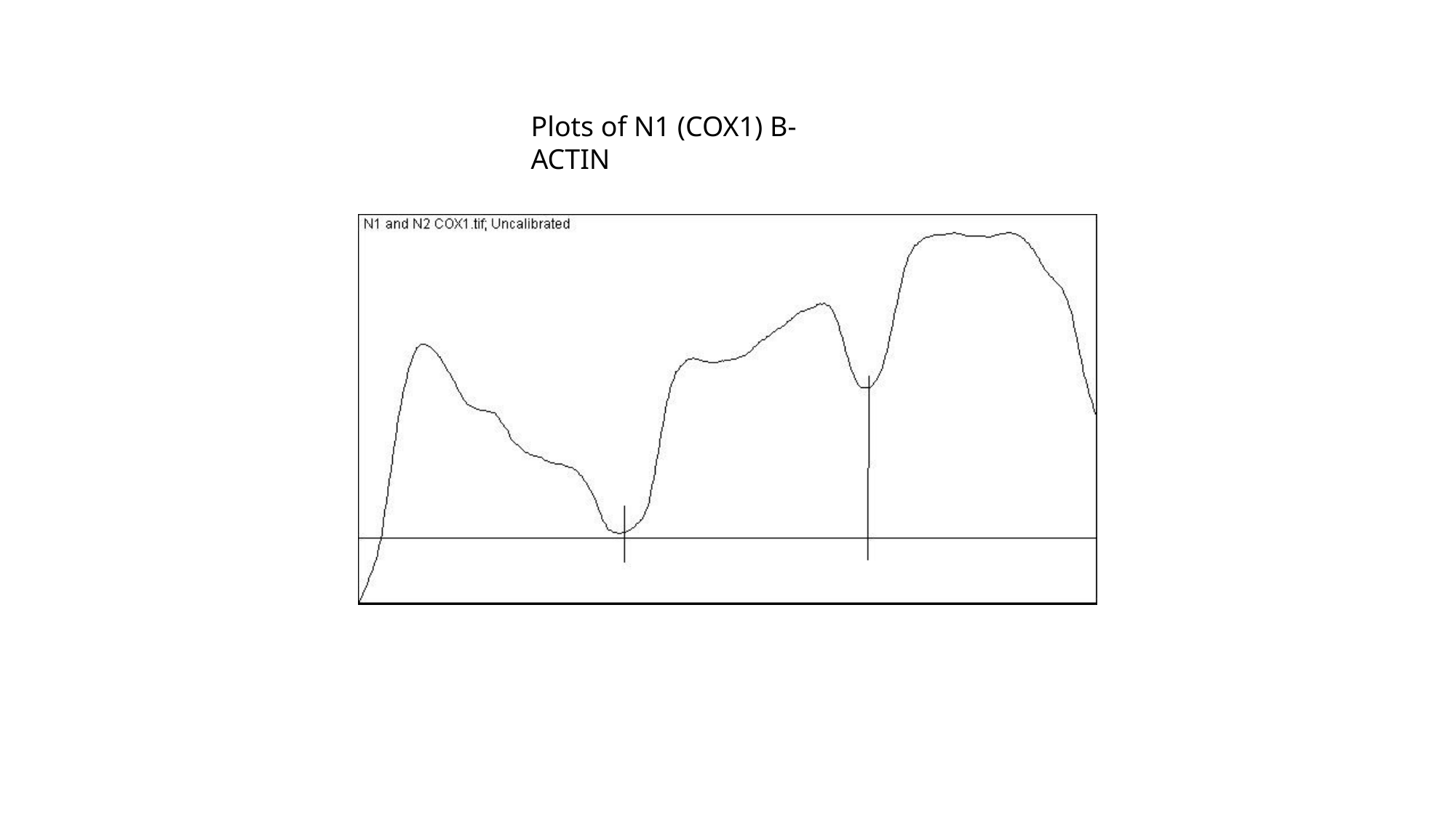

Plots of N1 (COX1) B-ACTIN

## Slide 3
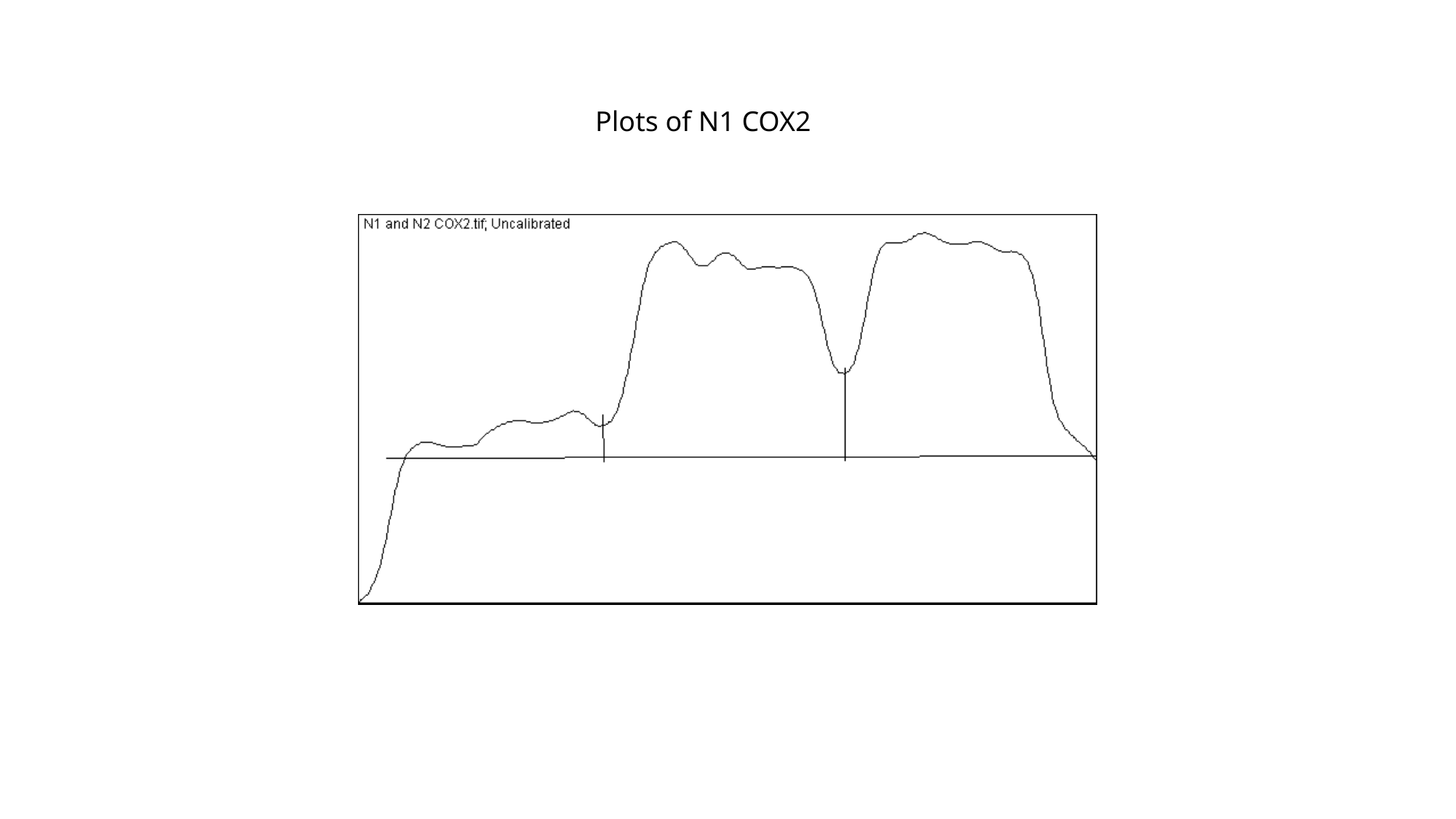

Plots of N1 COX2

## Slide 4
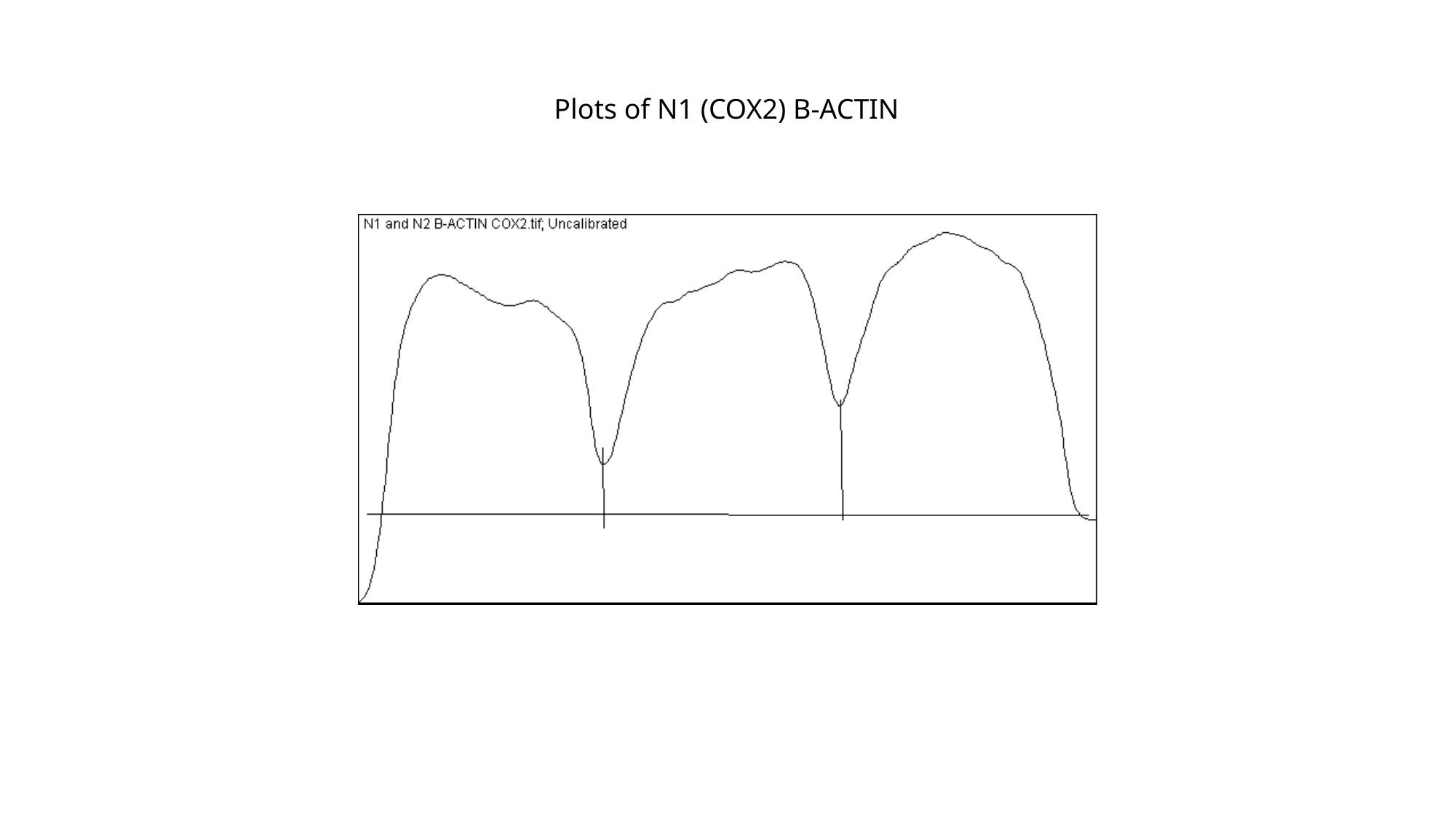

Plots of N1 (COX2) B-ACTIN

## Slide 5
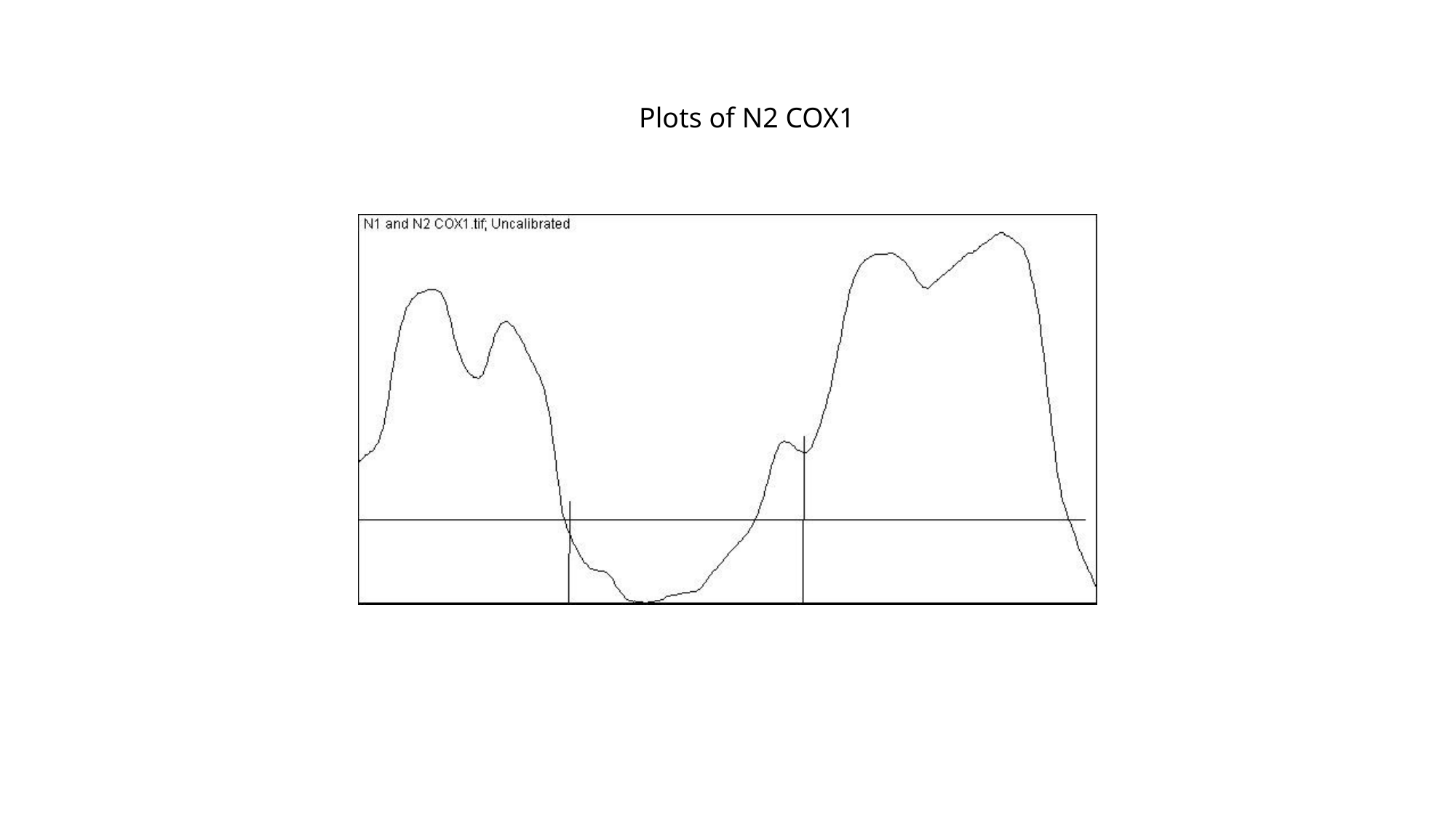

Plots of N2 COX1

## Slide 6
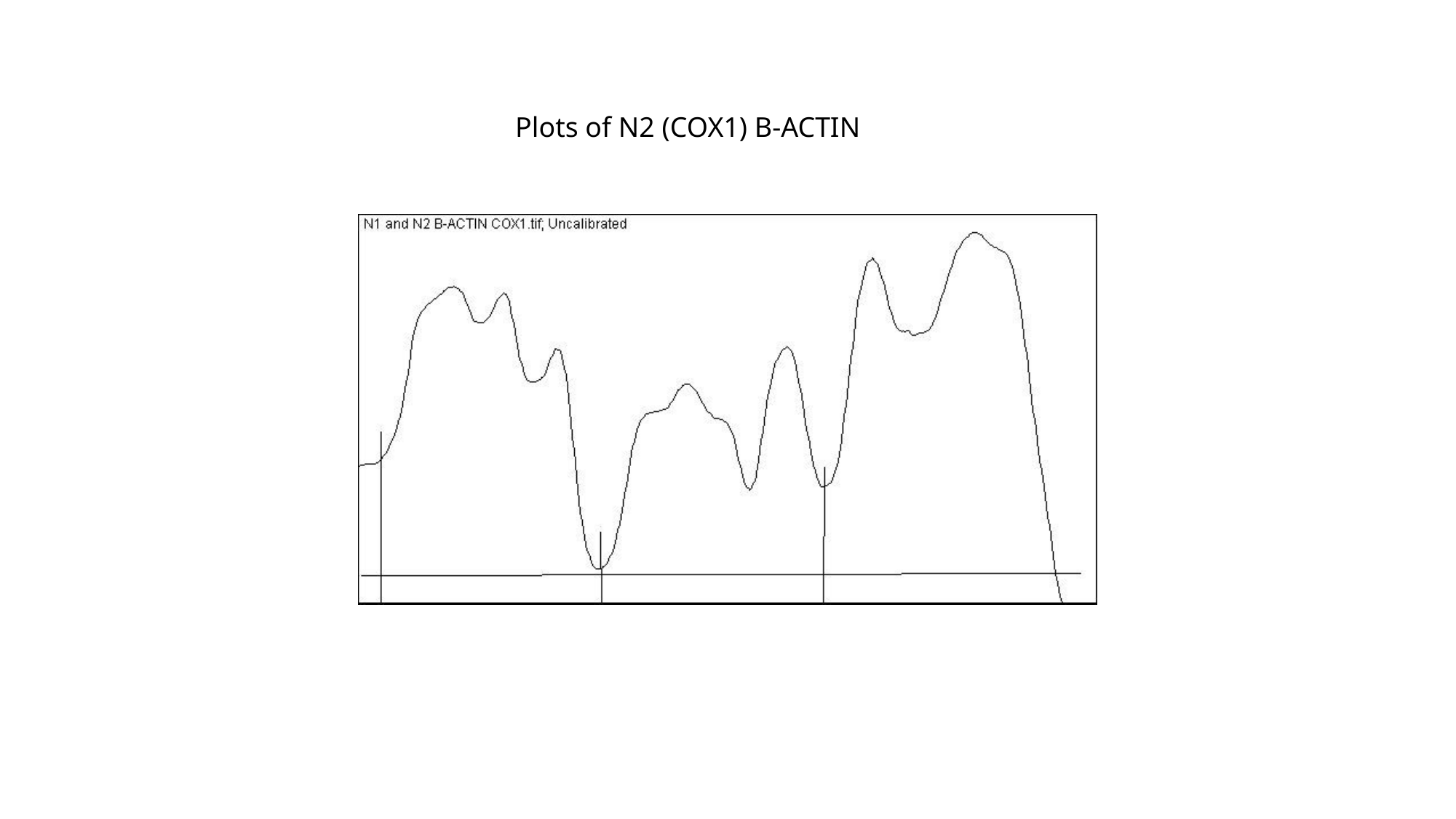

Plots of N2 (COX1) B-ACTIN

## Slide 7
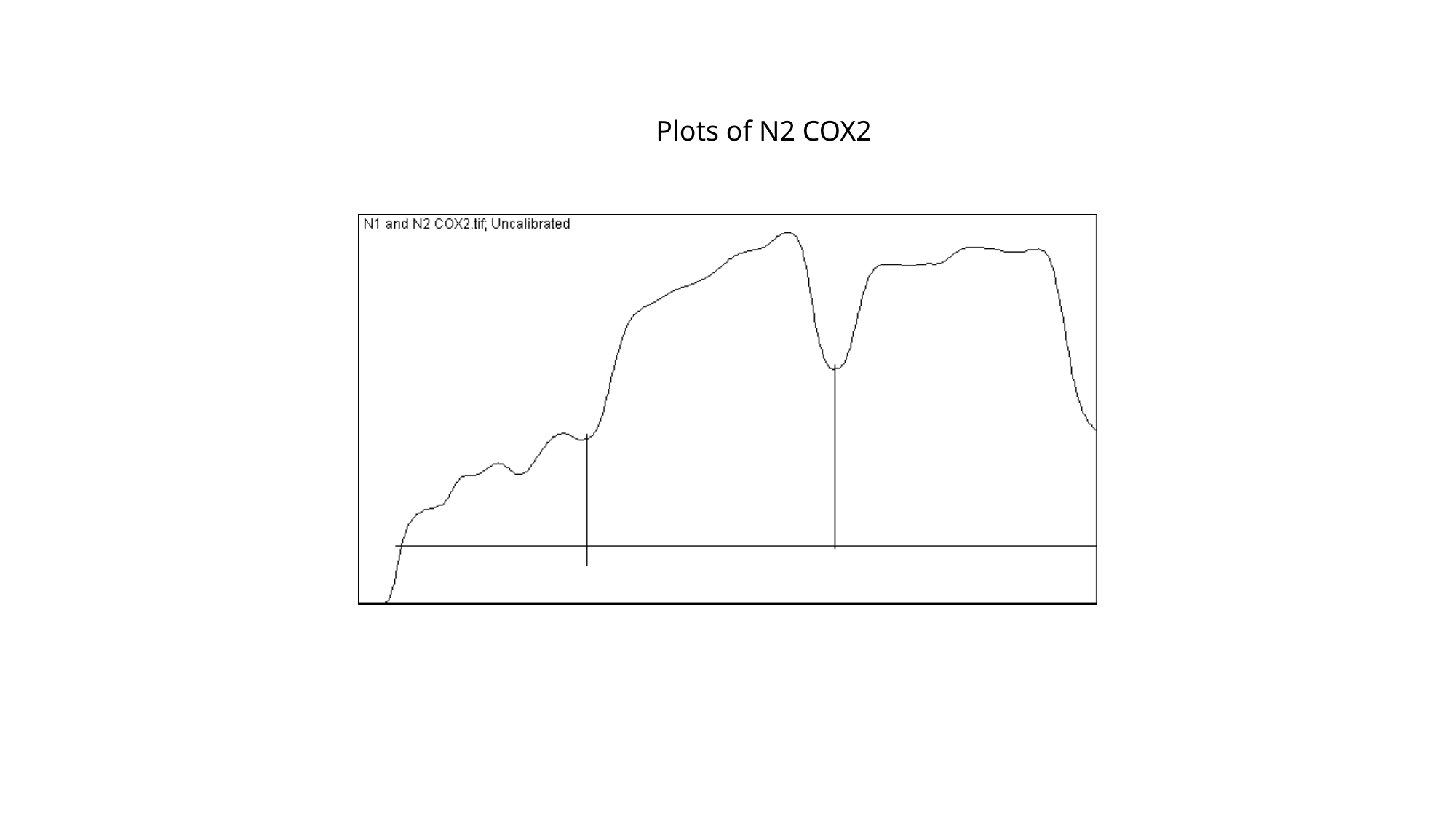

Plots of N2 COX2

## Slide 8
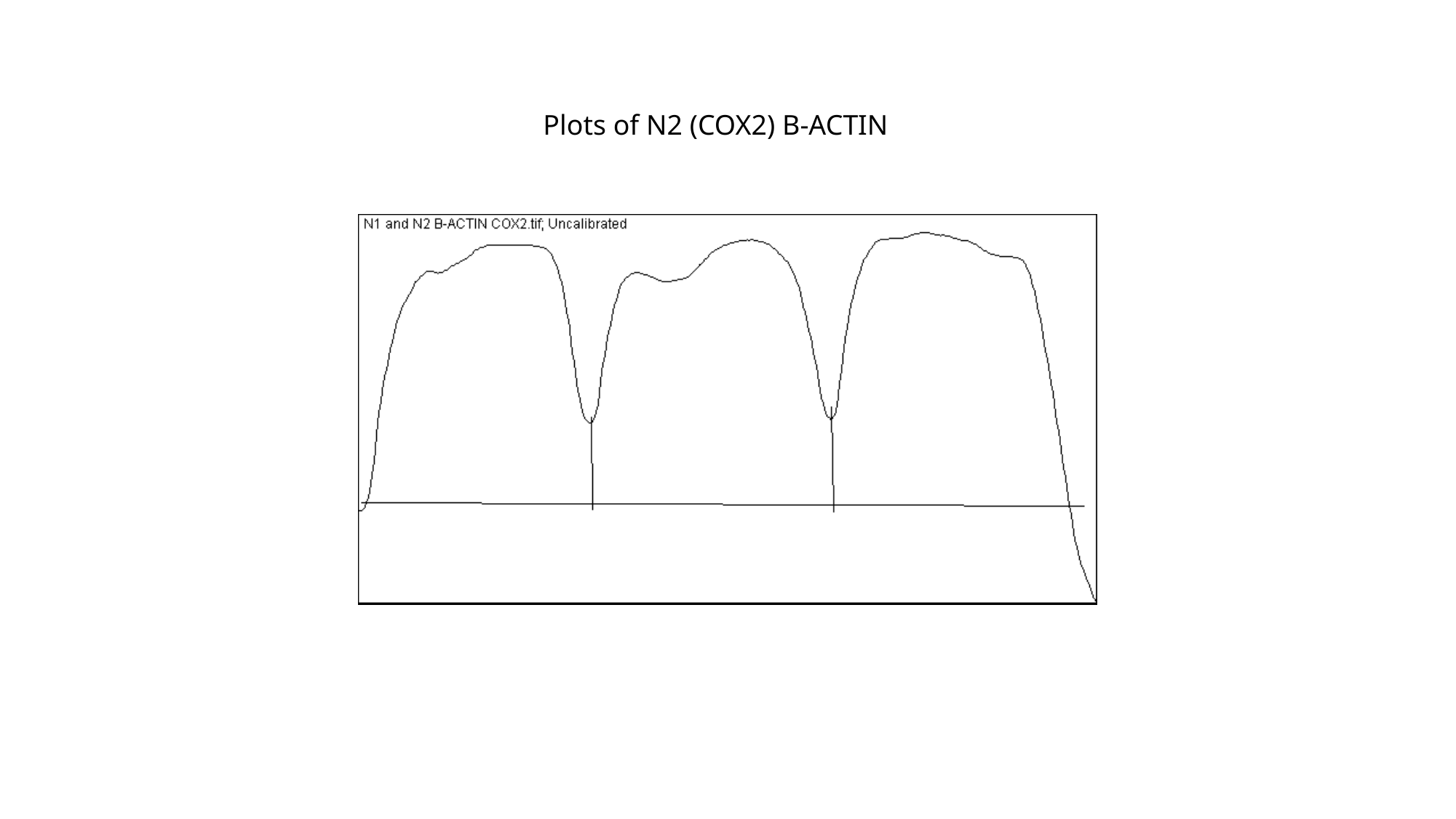

Plots of N2 (COX2) B-ACTIN

## Slide 9
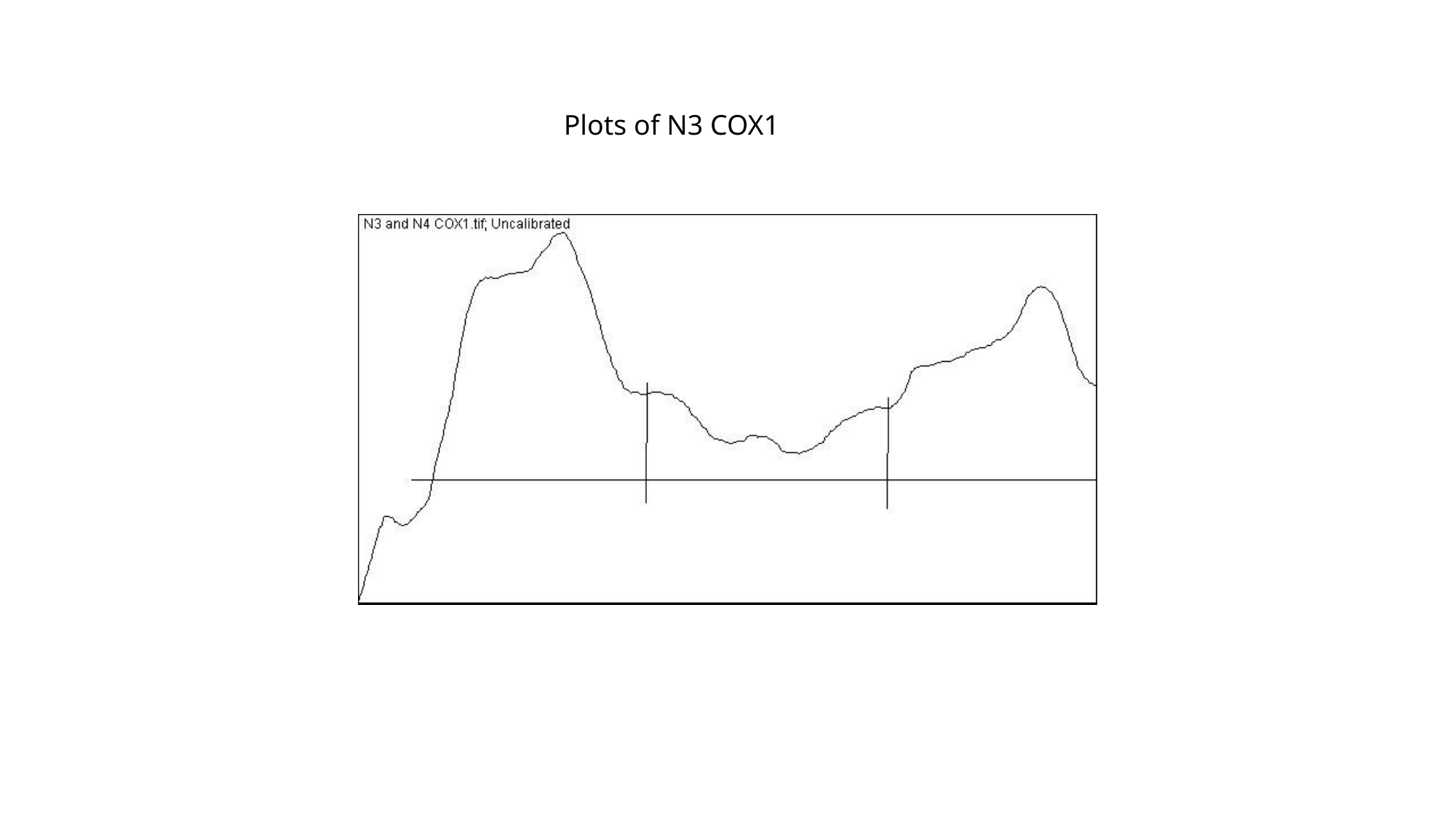

Plots of N3 COX1

## Slide 10
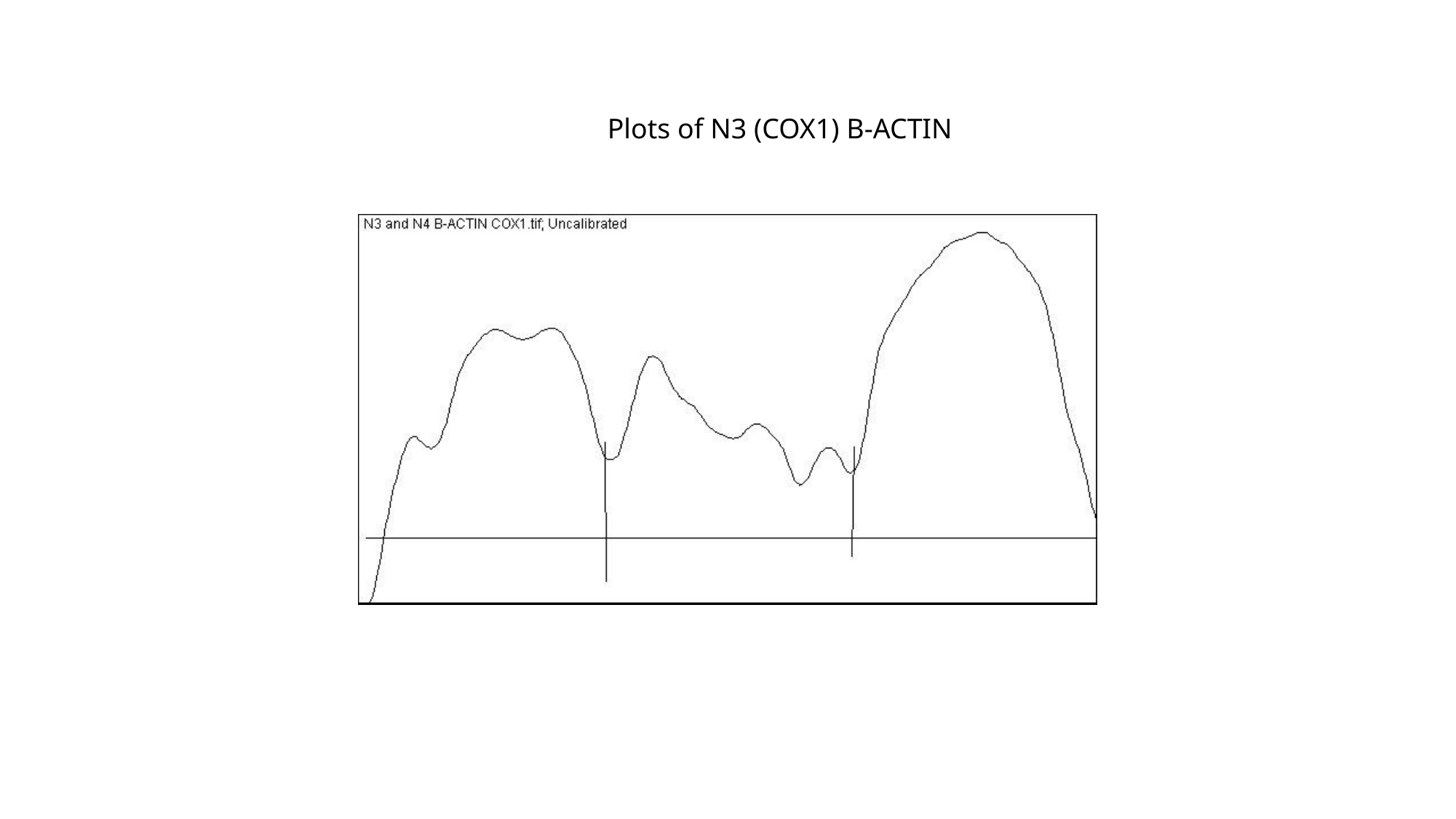

Plots of N3 (COX1) B-ACTIN

## Slide 11
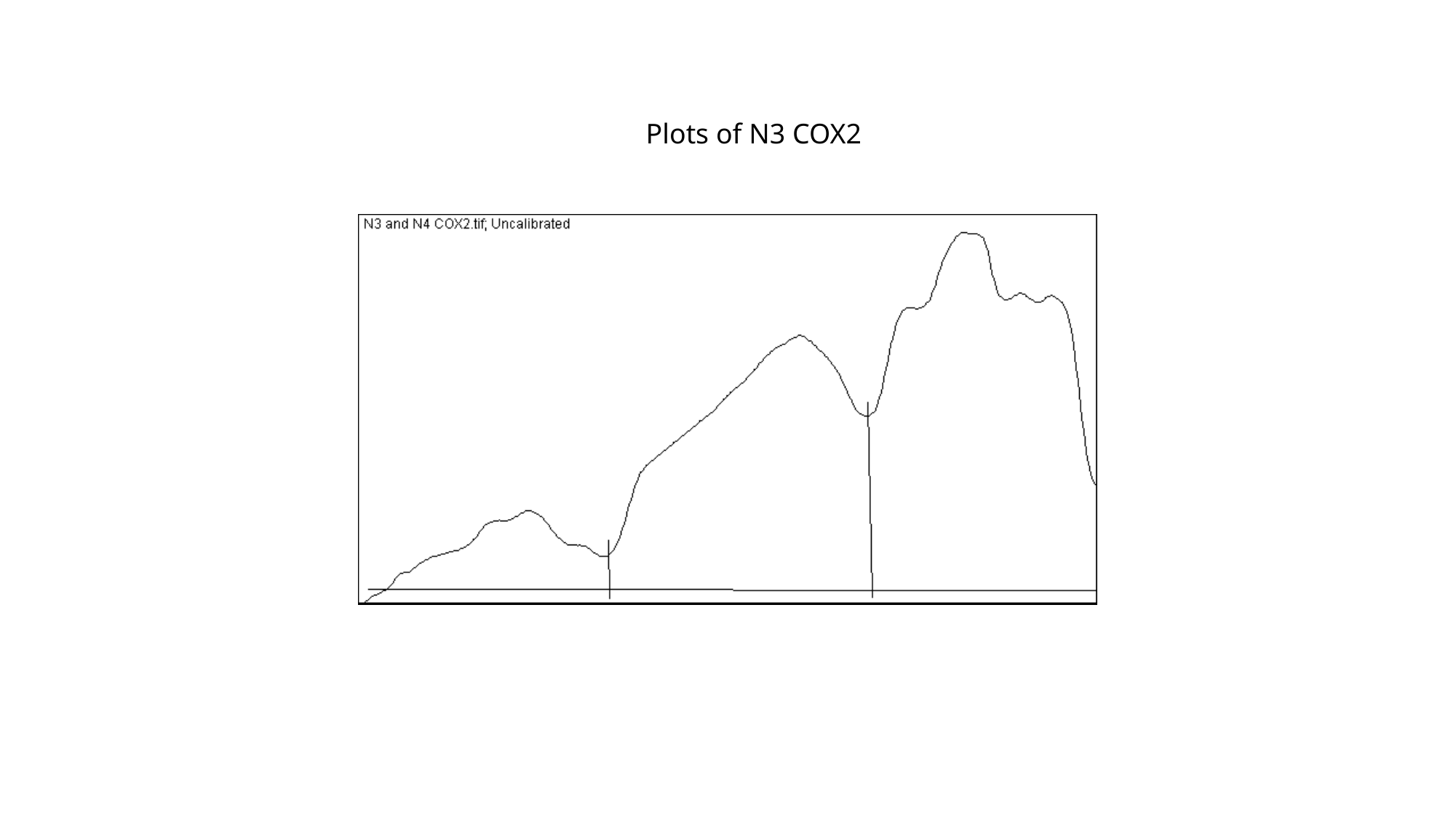

Plots of N3 COX2

## Slide 12
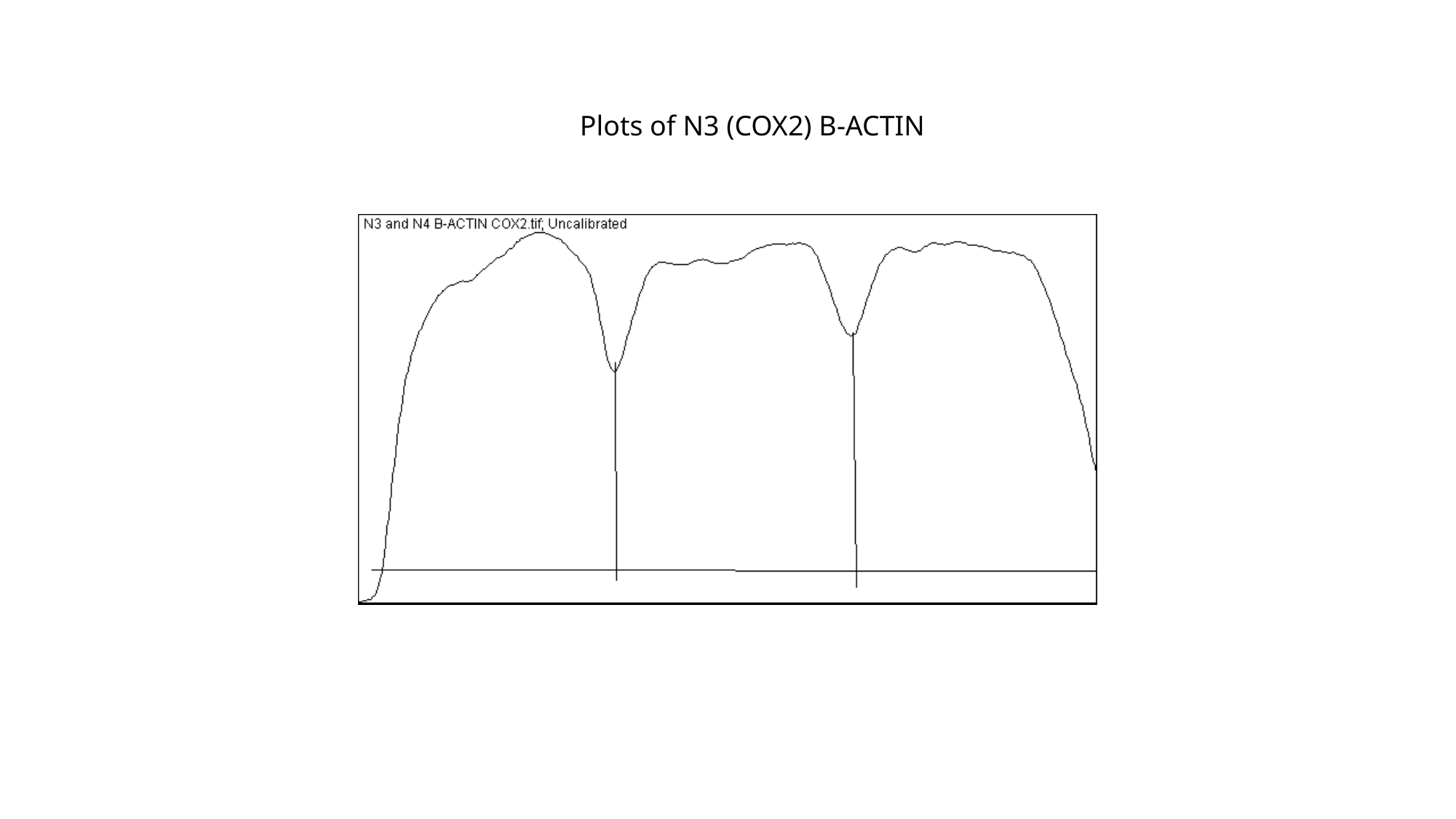

Plots of N3 (COX2) B-ACTIN

## Slide 13
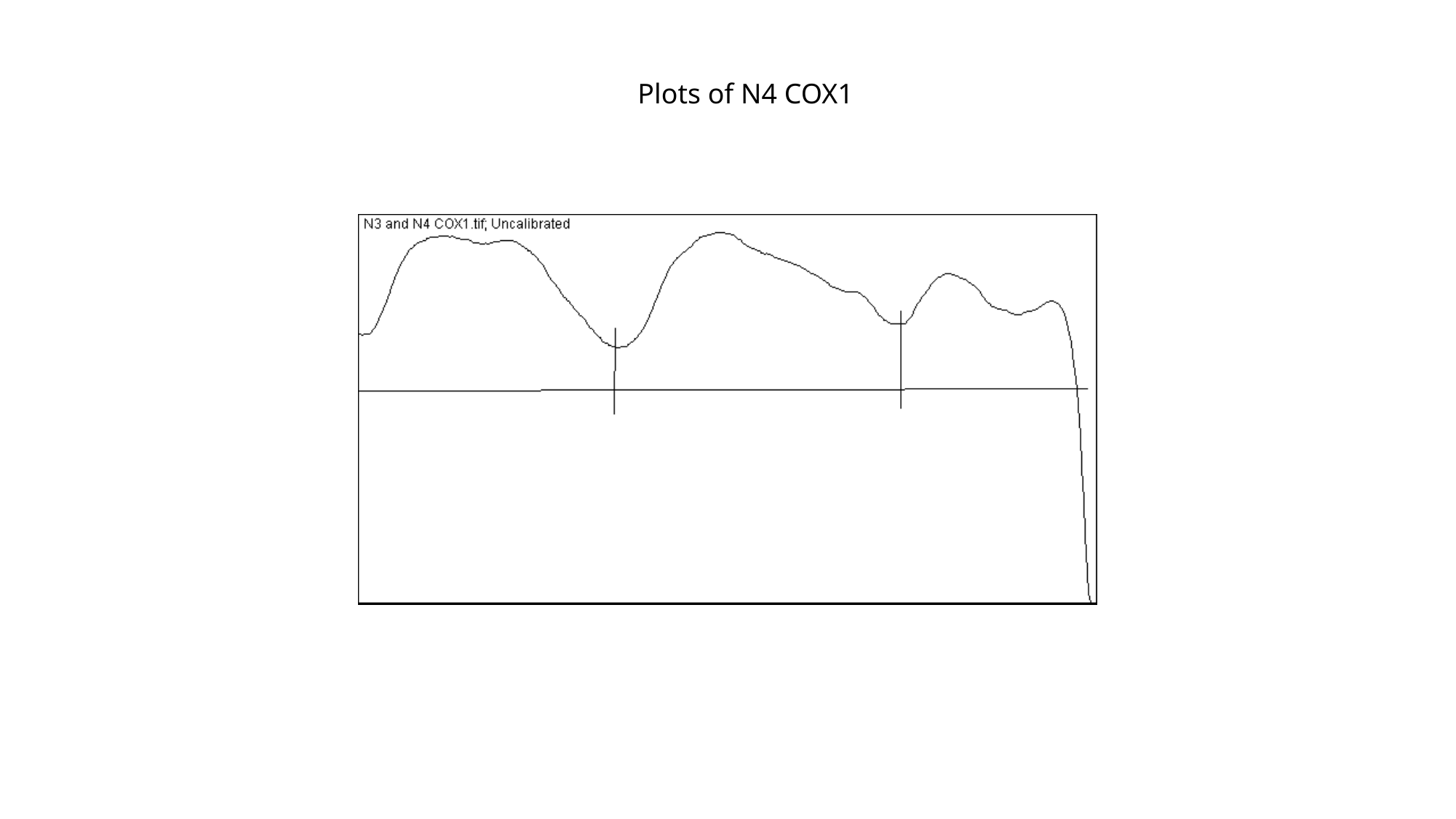

Plots of N4 COX1

## Slide 14
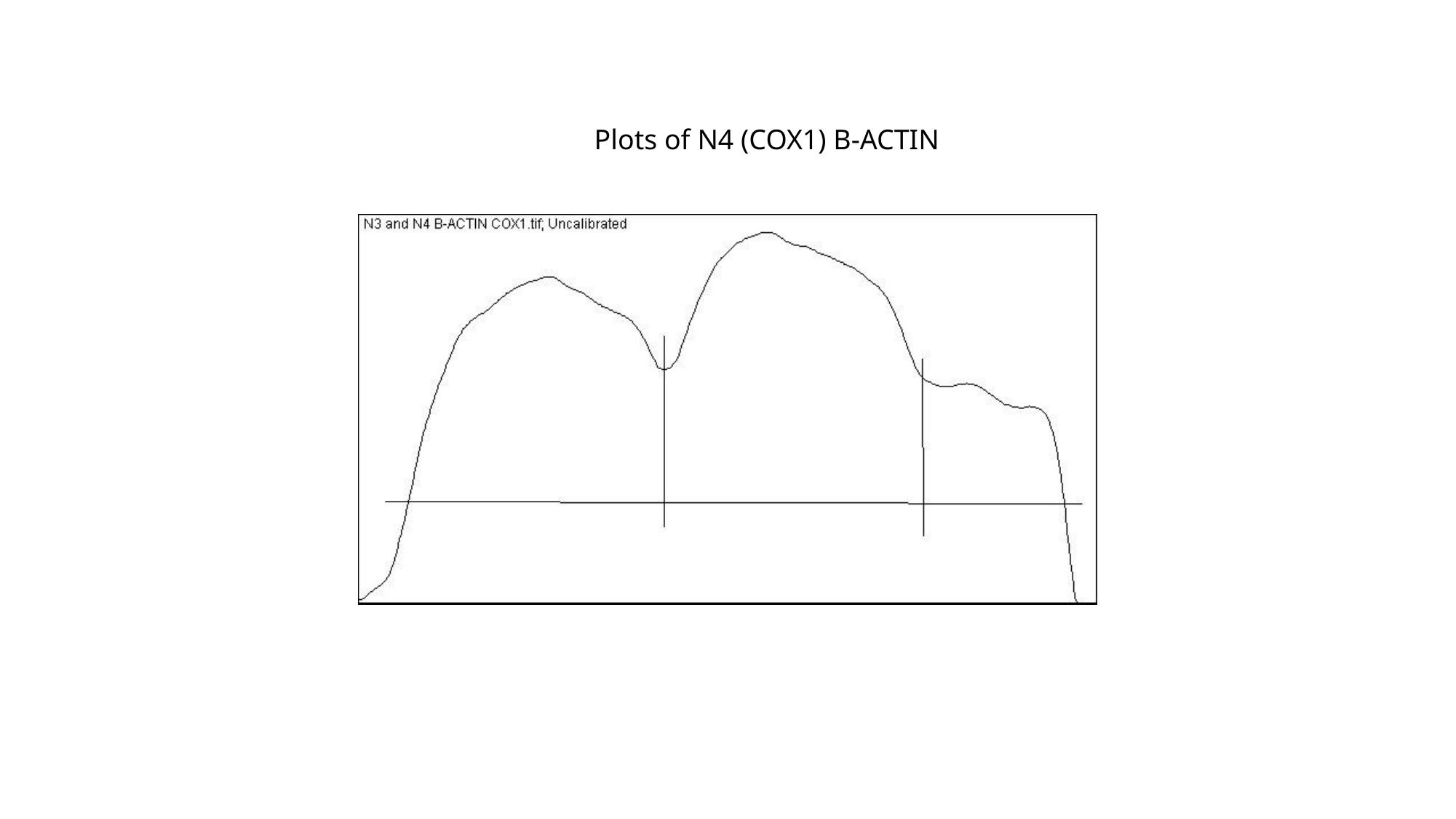

Plots of N4 (COX1) B-ACTIN

## Slide 15
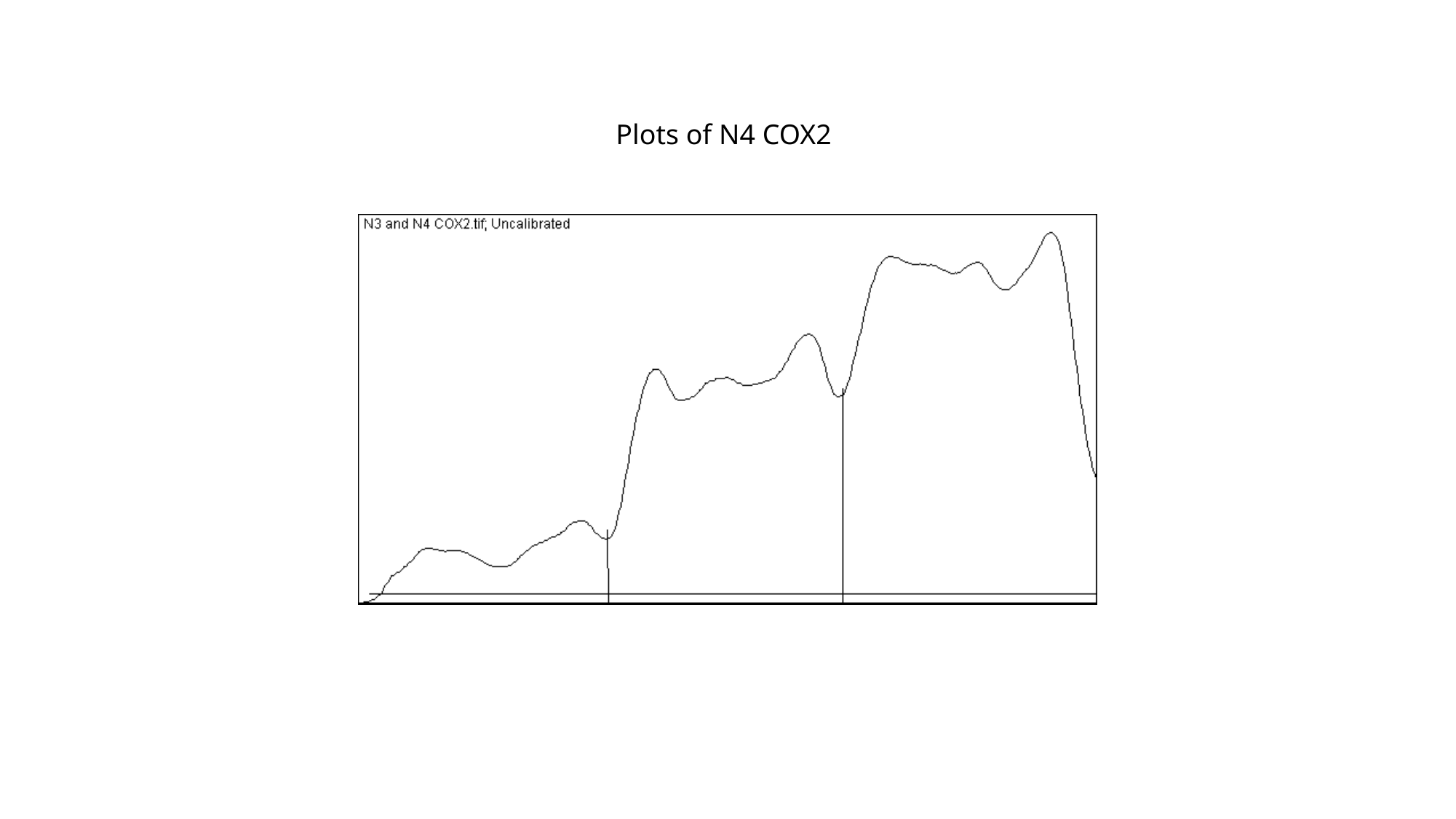

Plots of N4 COX2

## Slide 16
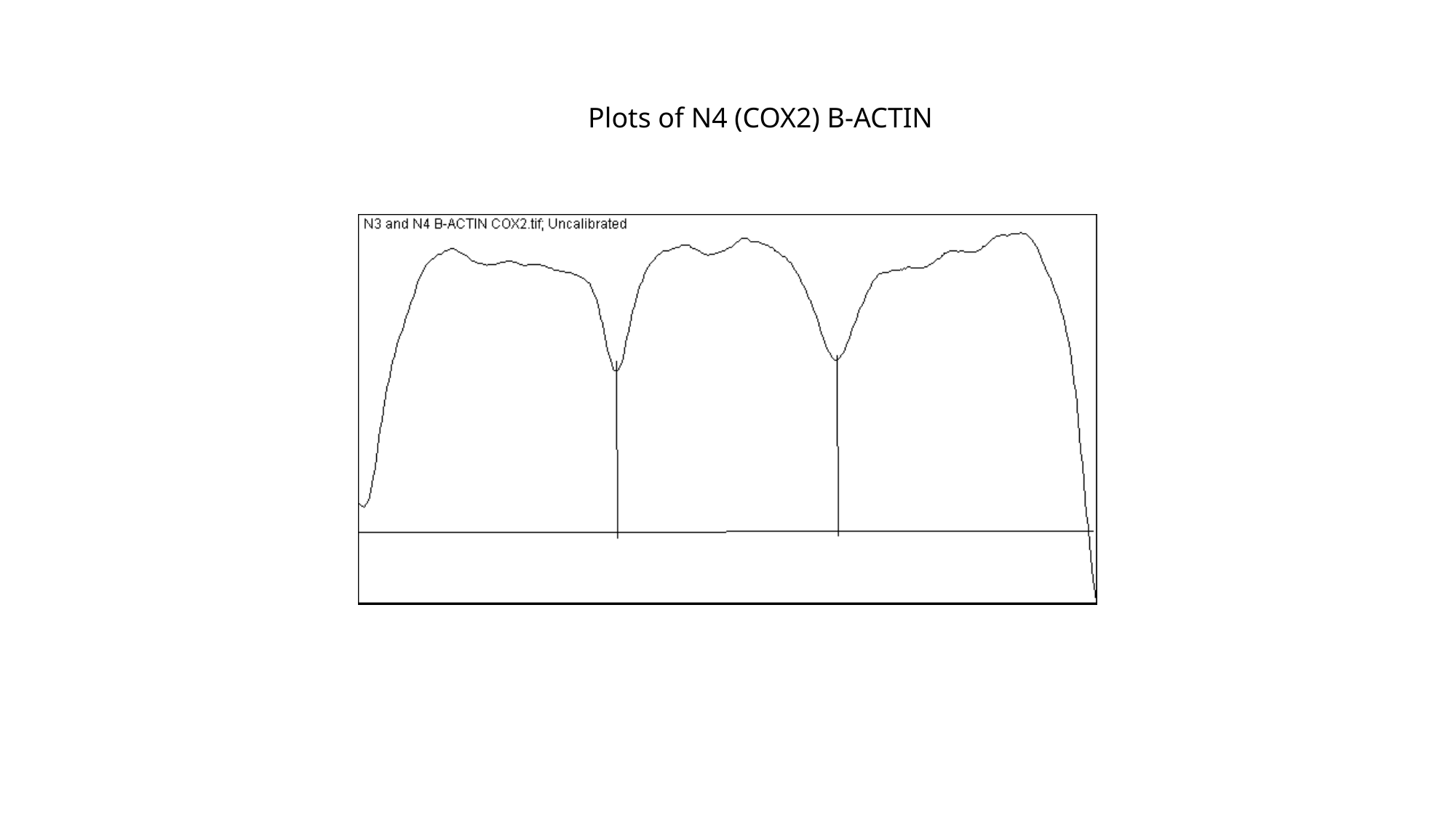

Plots of N4 (COX2) B-ACTIN
