# Supplementary figures and images for: Expression of Concern: Protective Role of Acetylsalicylic Acid in Experimental Trypanosoma cruzi Infection: Evidence of a 15-epi-Lipoxin A4-Mediated Effect
Source: PLoS Negl Trop Dis. 2024 Sep 5;18(9):e0012471. doi: 10.1371/journal.pntd.0012471 (PMC11376536; doi:10.1371/journal.pntd.0012471)

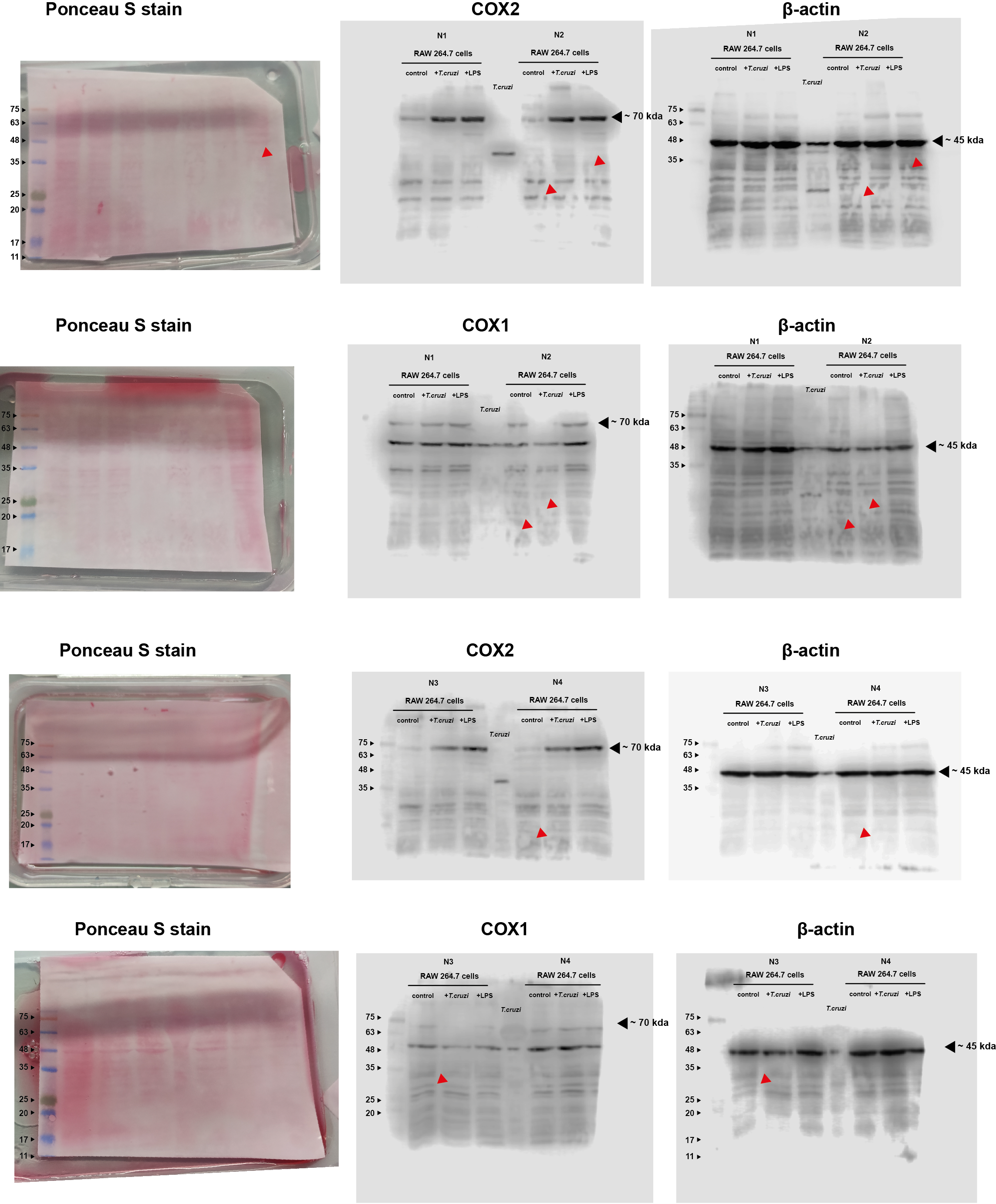

Supplement: S2 File — A) Original western blot images. B) Western blots with ponceau staining and molecular weight markers for experiment in S1 File. C) Quantitative data. D) Protocol including quantification method. E) Plots for density quantification. (ZIP) [file pntd.0012471.s002.zip › S2 File/B. Ponceau, Mol weight and line WB marks.tif]
